# Delusion-proneness displays comorbidity with traits of autistic-spectrum disorders and ADHD

**DOI:** 10.1371/journal.pone.0177820

**Published:** 2017-05-18

**Authors:** Anaïs Louzolo, Petter Gustavsson, Lars Tigerström, Martin Ingvar, Andreas Olsson, Predrag Petrovic

**Affiliations:** 1 Department of Clinical Neuroscience, Karolinska Institutet, Stockholm, Sweden; 2 Department of Neuroradiology, Karolinska Universitetssjukhuset, Stockholm, Sweden; University of California Los Angeles, UNITED STATES

## Abstract

There is an increasing body of evidence suggesting a significant comorbidity between psychotic disorders such as schizophrenia and attention-deficit/hyperactivity disorder (ADHD) or autism-spectrum disorders (ASD). Recently, research on psychosis-proneness in otherwise healthy individuals has been a promising way to better understand the mechanisms underlying psychosis. As both ADHD and ASD symptoms show a normal distribution in the general population, such trait comorbidity may confound studies on psychosis-proneness. Thus, understanding the extent to which psychosis-proneness relates to ADHD and ASD symptoms in healthy subjects is crucial for studies focusing on at-risk or psychosis-prone populations. In the present paper we tested the robustness of overlap between psychosis-proneness and ADHD/ASD symptoms, by studying correlations between the scores of three commonly-used questionnaires assessing delusion-proneness (Peters’ Delusion Inventory), ADHD tendencies (Adult ADHD Self-Report Scale) and ASD tendencies (Autism Quotient), on a large sample of healthy individuals (n = 925) using raw scores, prototypical questions and a factor analysis. The results showed consistently positive correlations between psychosis-proneness and ADHD-, as well as ASD-symptoms. While the effect was weak for ASD, it was moderate for ADHD. The findings support the idea that when investigating psychosis-proneness it is crucial to also take ADHD- and ASD-tendencies into account, in order to conclude that the reported results in a given study are specific to psychosis-proneness. The observed trait correlations also suggest a common pathway in the underlying information processing of these states.

## Introduction

Experimental behavioural and functional imaging research has recently begun to study delusion and psychosis-proneness[1–4]. This personality trait is related to psychotic disorders in that the risk to transit to a clinical psychotic disorder is increased in subjects with high degree of psychosis-proneness[5]. While this risk is substantially higher in a subset of individuals belonging to the ultra-high risk for psychosis group that is also is associated with other mental problems, many subjects with high delusion proneness do not have significant mental or health difficulties[6]. It has been suggested that some key aspects of information processing, such as predictive coding, may be shared between psychosis-proneness among healthy subjects and patients with a clinical psychotic disorder[1,3]. Thus, research on psychosis-proneness may increase the knowledge on the mechanisms involved in psychotic symptoms and psychotic disorders. Psychosis-proneness may also shed new light on why there is a transition from a trait to a clinical psychotic state. Moreover, otherwise healthy individuals with high psychosis-proneness are attractive to study since they have not had any antipsychotic treatment and no secondary chronic effects from the psychiatric disorder[7,8]. Although research on psychosis-proneness is a promising field that may increase the understanding of psychosis-related mechanisms in the brain it may also be confounded with traits related to other psychiatric disorders. The aim of this study is to test whether there is an association between psychosis-proneness and ADHD or autistic traits. From a mechanistic viewpoint, it is also of interest to understand the relationship between psychosis-proneness and other subclinical traits.

It has been suggested that schizophrenia is a chronic neurodevelopmental[9–11] psychotic disorder with a prevalence of about 1% in the general population[12]. It comprises both positive symptoms (including visual and/or auditory hallucinations, delusions, major thought disorders and paranoia) and negative symptoms (including social withdrawal, apathy and anhedonia) that often present a large diversity in terms of the level and patterns of cognitive dysfunctions. Schizophrenia patients also suffer from cognitive impairments leading to some disturbances in executive functions[13], working memory[14–16], and attention[17–19]. Unlike most neurodevelopmental disorders, schizophrenia is typically associated with a late onset of symptoms, around late adolescence or early adulthood while childhood onset schizophrenia is rare[20]. However, many individuals that will develop schizophrenia later on in life already have mental problems in childhood and adolescence[21].

Attention-deficit/hyperactivity disorder (ADHD) is a neurodevelopmental disorder with a childhood onset characterised by inattentive, hyperactive, and impulsive behaviours that are not found in typically developing children. There is a substantial overlap between schizophrenia and ADHD[22]. Children and adolescents with ADHD are more than 4 times likely to develop schizophrenia later in adulthood than healthy controls[23] and 5% of the ADHD patients were meeting criteria for schizophrenia[24]. Large epidemiological studies indicate that the co-occurrence of ADHD and schizophrenia is due to shared genetic factors, rather than representing completely aetiologically distinct subsyndromes[25].

Autism-spectrum disorder (ASD) is an early-onset neurodevelopmental disorder, characterised by repetitive patterns of behaviour, impairment in communication and social interactions that can also be associated with intellectual disabilities, language impairments, and impaired motor or attentive behaviours[26]. As for ADHD, it has been shown that ASD shares multiple phenotypic similarities and risk factors with schizophrenia and related disorders, and have been reported to co-occur at elevated rates[27]. For example, in a study by Stalhberg *et al*[24] it was found that 8% of their ASD patient population were meeting criteria for schizophrenia or another psychotic disorder. This percentage was increased to 15% when adding ASD patients presenting psychotic features co-occurring with bipolar disorder[24].

Both ASD-[28] and ADHD-symptomology[29,30] may be characterized by a normal distribution with sub-clinical symptoms that become clinical when there is a significant loss of function[31]. Thus, as for delusion-proneness, ASD and ADHD symptoms may be regarded as trait symptoms. Such subclinical symptoms in healthy subjects are characterized by similar behaviour[30] and underlying brain mechanisms[32] as in the categorically defined disorder.

Thus, although schizophrenia, ADHD and ASD are separate neurodevelopmental disorders with distinct clinical symptoms and features, an increasing number of studies are now pointing towards the existence of some overlap between these psychiatric disorders[33–35]. However, it is not known whether there is a similar comorbidity between the related traits (trait comorbidity), i.e. whether psychosis proneness is associates with increased sub-clinical ADHD or ASD symptoms. It is important to understand whether this relation exists, for several reasons: First, if subclinical psychosis related behaviours correlate with those two traits, studies focusing on psychosis and psychosis-proneness would need to take ADHD and ASD tendencies into account, in order to validate that the reported results are specific to psychosis-proneness. Second, if there is a relation between these traits, mediation and moderator effects of ADHD and ASD traits on the psychoses or psychosis proneness may be studied.

In the current study we asked a large sample of healthy male individuals to complete three commonly-used questionnaires assessing delusion-proneness, ADHD symptoms and ASD symptoms (i.e. Peters’ Delusion Inventory/PDI[36], Adult ADHD Self-Report Scale/ASRS[37] and Autistic disorder Quotient/AQ[38], respectively). We then examined the relationships between PDI scores and the two other questionnaires. We were especially interested in the relationships between the questions/statements that were most specific to the different disorders, i.e. prototypical, and performed factor analysis on these items. Our hypothesis was that PDI scores would correlate positively with ASRS and AQ scores, and these relations would remain when solely taking into account the prototypical statements (i.e, when associations are not reflecting item overlap). Namely, we hypothesized that the more delusion-prone the participants were, the stronger their ADHD and ASD tendencies would be. We also hypothesized that a factor analysis on these items would identify the main factors related to the different traits and that these different factors also would be related to each other. Such findings would suggest comorbidity between these traits and would require to investigate ADHD and ASD symptom levels in studies focusing on psychosis-proneness. Moreover, it would suggest a relation between the underlying mechanisms involved in these traits.

## Methods

### Participants

We screened 925 male individuals aged 18 to 35 years (mean 24.98 years, SD 0.161) for delusion proneness, ADHD- and ASD-tendencies, using online versions of the Peters’ Delusion Inventory (PDI—21 items)[36], the World Health Organization Adult ADHD Self-Report Scale (ASRS), and the Autism Spectrum Quotient questionnaire (AQ)[38]. We specifically targeted male individuals as, depending on their scores on the questionnaires, some of the participants then took part in an experimental study that required only male participants. Subjects were recruited through advertising services on social media reaching a diverse study population. The target audience of the advert (in other words, people who saw the advert in their newsfeed) were male individuals aged 18 to 35, living in the Stockholm area (city centre +40km around). Since all the questionnaires were in Swedish, the advert (written in Swedish) also specified participants had to be native speakers. The subjects did not provide information about their education or socioeconomic background. It was stressed twice that they had to be healthy and without any psychiatric diagnosis. Upon submission of their contact details and after giving their consent to take part in this study, participants received a link to the questionnaires and an automatically-generated unique ID-code they used when filling in the questionnaires. The study was approved by the regional ethical board of Stockholm (Regionala Etikprövningsnämnden i Stockholm—Dnr 2012/1701-31/3).

### Questionnaires

We made an online adaptation of the 3 questionnaires. The name of each questionnaire was removed but the instructions were displayed as in the original paper version.

#### 1) 21-item Peters et al. Delusions Inventory (PDI-21)

PDI-21 is a self-administered questionnaire developed by Peters and colleagues as a schizotypal scale measuring delusional ideation in the general population[36]. The questionnaire comprises 21 questions assessing a wide range of possible delusions. Subjects should indicate whether each statement applies to them. Answering “yes” to a question gives 1 point, leading to a maximum global score of 21 points (this score will be referred to as *yes/no score*). In addition, for each item endorsed, three dimensions have to be rated on a 5-point Likert scale (1–5), in order to assess how convinced, distressed, and preoccupied by the delusion the participant is (i.e. *conviction*, *distress*, and *preoccupation* scores, respectively). Since the total score on those three dimensions is dependent on the number of endorsed beliefs, we computed relative sub-scores by dividing the total value of each of the three dimensions (*conviction*, *distress*, *preoccupation*) by the total number of endorsed items (*yes/no score*). These will be referred to as *conviction sub-score*, *distress sub-score* and *preoccupation sub-score* in the remaining of the paper. The use of such relative sub-scores has been described previously[39].

#### 2) The World Health Organization Adult ADHD Self-Report Scale (ASRS)

ASRS is a self-report screening scale developed by the World Health Organization (WHO). ASRS was developed in conjunction with revision of the WHO Composite International Diagnostic Interview (CIDI)[37], in order to measure ADHD tendencies and to be used as a part of a clinical ADHD-assessments in adult individuals. The questionnaire contains 18 questions, and using a 5-point Likert scale, participants have to report how frequently a particular symptom of ADHD has occurred to them over the past six months. The responses range from never (0), rarely (1), sometimes (2), often (3), to very often (4), to a total score range from 0 to 72. The questionnaire can be divided into two part (part A/part B scoring) in order to use it as a screening tool. However in the present study we used the full questionnaire (18 questions) and we ran our analyses on the summed frequency scores (referred to as the total ASRS score). We only used the part A/B screening in a sub-analysis, in order to exclude individuals that possibly show relevant symptoms.

#### 3) Autism-spectrum quotient (AQ)

AQ is a self-administered questionnaire developed in order to assess the degree to which an adult individual is displaying autistic traits[38]. The questionnaire comprises 50 questions that can be grouped into five subscales (10 questions each) assessing: social skills, attention switching, attention to detail, communication and imagination. Participants rate how much they agree or disagree on the different statements (habits and personal preferences)[38]. Each item scores 1 point if the participant selects the behaviour more often observed in autistic individuals, giving a maximum score of 50 points; higher scores indicate stronger autistic-like behaviour tendencies[38]. Baron-Cohen *et al* suggested that a score above 32 is a useful cut-off in order to distinguish individuals with a clinically significant levels of autistic traits[38].

The distributions we obtained on the three questionnaires (PDI-21 total score, AQ total scores and ASRS total scores) in the present study, were quite similar to what is commonly reported (Table 1). The three questionnaires showed an acceptable to good internal consistency: AQ (α = 0.788), and PDI-21 yes/no score α = 0.772 (distress α = 0.816, preoccupation α = 0.816, conviction α = 0.791) and ASRS α = 0.862.

**Table 1:** Distributions of the scores on AQ, ASRS (total score, ASRS-inattention and ASRS- hyperactivity/impulsivity), PDI (yes/no, and three subscores).

	Mean *(n = 925)*	S.D.
AQ	15.79	6.352
ASRS total	29.78	10.355
ASRS inattention	15.69	5.989
ASRS hyperactivity/impulsivity	14.09	5.779
PDI yes/no	5.87	3.724
PDI distress subscore	13.95	11.702
PDI preoccupation subscore	15.4	12.084
PDI conviction subscore	17.91	13.016

### Data analysis

In order to investigate how psychosis-proneness relates to ADHD and ASD, we studied the correlations between the PDI-21 yes/no score, and the total scores for ASRS and AQ, using Spearman’s rank correlations. As an exploratory analysis, we also studied the relation between the PDI-21 yes/no score and the three sub-scores (conviction, preoccupation and distress).

We performed a factor analysis over all the items of the questionnaires in order to study how the three disorders were related, and more specifically to which aspects of ADHD and ASD was delusion-proneness the most related. This factor analysis followed the procedure outlined for exploratory structural equation modelling (ESEM)[40], applying an exploratory search for a restricted number of factors, and a confirmatory approach to test the fit of the model. Since we assumed that each questionnaire would represent at least one factor, we first conducted our analysis using a 3-factor model. Depending on the fit of the model we planned also to test for more explanatory factors until acceptable model fit was reached. In a final step we studied the correlation between the factors of the best fitting model. Item response data was treated as ordered categorical data and analyses were performed using polychoric correlations and weighted least squares estimation in the Mplus 7.2 program[41]. Following the rules of thumb for interpreting fit indices, criteria for an acceptable model fit was an RMSEA under 0.06 and a CFI over 0.95[42]. We also perform correlation analyses between the different factors.

Some of the questions in the three questionnaires are not prototypical to the disorder that is supposedly assessed in a given questionnaire. Instead they can also be good descriptors of other psychiatric states or traits. Indeed, as evidenced by the first factor analyses, when using the full version of the questionnaires, some items of AQ and ASRS load onto the same factors. However, some items loaded on factors that are not usually considered characteristic of one of the two disorders. For instance, Factor 1 comprises ASRS items that are clearly associated with impulsivity while it also contains AQ items that do not seem so closely related to impulsivity. In addition, ASD is not usually considered to be associated with impulsivity behaviours. In a second step we thus tried to minimize this problem by only including items that were the most specific to autism, ADHD, and psychosis in AQ, ASRS, and PDI, respectively, hoping to obtain more clearly-defined factors. In order to select those items, two psychiatrists rated how prototypical each question/statement was to each of the three different disorders based on diagnostic criteria and their clinical experience. In the selection process to find the most prototypical items all the questions/statements were mixed across the three questionnaires and then rated from 0 to 10 by the two psychiatrists. We averaged their ratings and for each of the three questionnaires we then defined the item as prototypical if it had a rating of 7 and above for the disorder they were supposed to characterise, and a difference of 5 points or more compared to the two other disorders. For example, if an ASRS question was rated 8 on ADHD, 2 on autism and 3 on psychosis, it was selected, while if another ASRS question was rated 8 on ADHD, 3 on autism but 4 on psychosis, it was disregarded. This led to the selection of fourteen questions out of fifty in AQ, thirteen out of eighteen in ASRS, and nine out of twenty-one in PDI-21 (see S2 Table) that were defined as prototypical. There was a low to acceptable internal consistency for these prototypical versions of ASRS (α = 0.821), AQ (α = 0.591), and PDI-21 (yes/no score α = 0.662, Distress sub-score α = 0.682, Preoccupation sub- score α = 0.705, Conviction sub-score α = 0.679). The drop in internal consistency in the AQ, and to a lesser extent PDI, was due to the fact a large number of items that were removed from the original AQ (36 out of 50) and PDI (more than half of the total items). We then performed correlation analyses on the truncated version of each questionnaire.

Then, in order to have a clearer picture of which ADHD- and ASD-features, delusion-proneness is the most closely associated with, we decided to also perform a factor analysis on the selected prototypical questions/statements (PDI-21 yes/no score, AQ score and ASRS score). We used the same approach as with the full versions of the questionnaires.

## Results

### Relation between total scores of the full versions of PDI and ASRS / AQ

**Fig 1:**
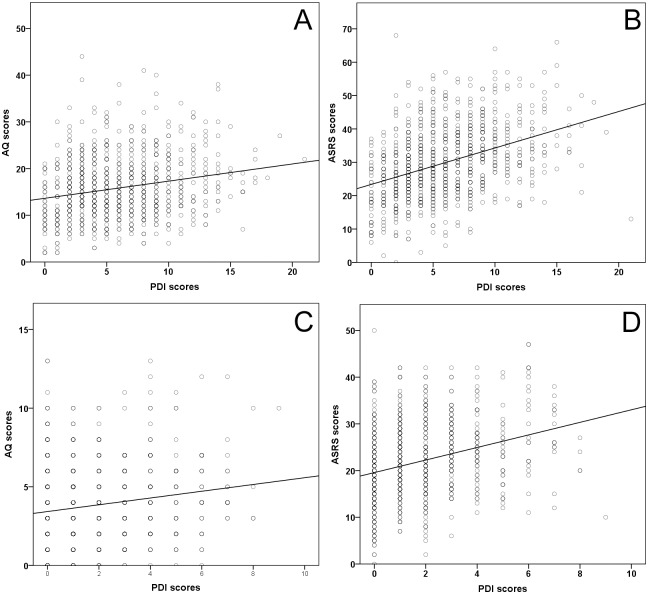
Correlations between PDI scores and AQ/ASRS scores on full and truncated versions of the questionnaires—two-tailed Spearman’s correlations—** p<.001. **A**: Correlation between PDI scores and AQ scores (full questionnaires)—r = 0.192**. **B**: Correlation between PDI scores and ASRS scores (full questionnaires)—r = 0.406**. **C**: Correlation between PDI scores and AQ scores (truncated questionnaires)—r = 0.129**. **D**: Correlation between PDI scores and ASRS scores (truncated questionnaires)—r = 0.324**.

Spearman’s rank correlations between PDI-21 yes/no scores and AQ total scores showed a weak effect (r = 0.192, p<0.001 –two-tailed) (Fig 1A). The same test suggested a moderate correlation between PDI-21 yes/no scores and ASRS total scores (r = 0.406, p<0.001 –two-tailed) (Fig 1B and S1 Appendix for correlations between PDI scores and ASRS scores divided into inattention and hyperactivity/impulsivity scores). Since our prime interest was delusion-proneness, we had to make sure those correlations were not driven by individuals displaying a possible ASD and/or ADHD symptomatology. We thus performed the same tests after removing individuals who scored above the conventional screening threshold in AQ (score > 32) and/or ASRS (score ≥ 4 on ASRS-part A with the part A/part B scoring method). Even without these individuals (26.6% of the total sample), we still observed similar correlations (PDI-AQ correlation: r = 0.190, p<0.001 –two-tailed; PDI-ASRS correlation: r = 0.345, p<0.001 –two-tailed, n = 672). PDI relative sub-scores Distress and Preoccupation were moderately related to PDI-21 yes/no scores (r = 0.454, p<0.001 and r = 0.447, p<0.001 respectively) while this relation was weak for the conviction sub-score (r = 0.225, p<0.001).

### Factor analysis using full versions of PDI and ASRS / AQ

We started the factor analyses using a 3-factor model, however the fit was not satisfactory. Due to the large number of items in our dataset we had to increase the number of factors to seven in order to reach a good fit to the data (Table 2 and S3 Table). This model had an RMSEA of 0.019 and a CFI of 0.943. Factor 1 and 6 contained a mix of ASRS and AQ items. Factor 1 (denoted *Social Interaction Impairment factor*), Factor 2 (*Focus on details* factor), and Factor 4 (*Theory of mind*) comprised respectively twelve, seven and seven AQ items, with standardised loading estimates of at least 0.4. Factor 3 (*Communication/Social impulsivity factor*) comprised three AQ item and four ASRS items. Factor 5 contained fifteen items from PDI-21 (*Delusion-proneness factor*). Factor 6 (*Inattention factor*) and Factor 7 (*Hyperactivity factor*) comprised respectively, six and five items, all from ASRS. When performing a correlational analysis between the factors, we found that the *Delusion-proneness factor* was significantly correlated with four of the seven factors (see Table 3 and S4 Table); three of the four factors were more related to ADHD-like symptoms, while the fourth one was closer to an ASD-like dimension.

**Table 2:** 7-factor model (full questionnaires).

		Two-Tailed	
Factors	Item	Estimate	p-value
Factor 1—Social Interaction Impairment	AQ01	0.508	0.000
	AQ11	0.821	0.000
	AQ13	0.554	0.000
	AQ15	0.609	0.000
	AQ17	0.807	0.000
	AQ22	0.666	0.000
	AQ26	0.684	0.000
	AQ34	0.516	0.000
	AQ38	0.836	0.000
	AQ44	0.91	0.000
	AQ46	0.554	0.000
	AQ47	0.917	0.000
Factor 2—Focus on details	AQ05	0.419	0.000
	AQ06	0.536	0.000
	AQ09	0.453	0.000
	AQ12	0.619	0.000
	AQ19	0.561	0.000
	AQ23	0.578	0.000
	AQ29	0.485	0.000
Factor 3—Communication/Social Impulsivity	AQ07	0.462	0.000
	AQ18	0.753	0.000
	AQ39	0.51	0.000
	ASRS15	0.568	0.000
	ASRS16	0.405	0.000
	ASRS17	0.57	0.000
	ASRS18	0.479	0.000
Factor 4—Theory of mind	AQ03	0.418	0.000
	AQ08	0.544	0.000
	AQ20	0.624	0.000
	AQ27	0.644	0.000
	AQ31	0.41	0.000
	AQ36	0.635	0.000
	AQ45	0.716	0.000
Factor 5—Delusion-proneness	PDINY02	0.472	0.000
	PDINY04	0.557	0.000
	PDINY05	0.469	0.000
	PDINY06	0.567	0.000
	PDINY07	0.437	0.000
	PDINY08	0.74	0.000
	PDINY09	0.541	0.000
	PDINY11	0.778	0.000
	PDINY12	0.614	0.000
	PDINY15	0.418	0.000
	PDINY17	0.521	0.000
	PDINY18	0.419	0.000
	PDINY19	0.549	0.000
	PDINY20	0.488	0.000
	PDINY21	0.463	0.000
Factor 6—Inattention	ASRS01	0.503	0.000
	ASRS02	0.637	0.000
	ASRS03	0.474	0.000
	ASRS04	0.629	0.000
	ASRS08	0.411	0.000
	ASRS10	0.437	0.000
Factor 7—Hyperactivity	ASRS05	0.531	0.000
	ASRS06	0.762	0.000
	ASRS08	0.402	0.000
	ASRS13	0.818	0.000
	ASRS14	0.479	0.000

List of items with standardised loadings of at least 0.4, on each of the seven factors

**Table 3:** Significant correlations between the PDI-factor and four factors from the 7-factor model analysis (full questionnaires).

		Two-Tailed	
		Estimate	p-value
Delusion-proneness with	Focus on details	0.152	0.000
	Communication/Social impulsivity	0.240	0.000
	Inattention	0.176	0.000
	Hyperactivity	0.244	0.000

### Relation between truncated versions of PDI and ASRS / AQ

We also studied the relation between PDI and ASRS/AQ using only the selected disorder-specific (i.e. prototypical) items in the three questionnaires. These tests showed that PDI-21 yes/no scores were still correlating weakly with AQ scores (r = 0.129 p<0.001 –two-tailed Spearman’s correlation) (Fig 1C) and moderately with ASRS scores (r = 0.324 p<0.001 –two-tailed Spearman’s correlation) (Fig 1D). Thus, the relation between delusion-proneness and ADHD/ASD traits remained after partially controlling for less specific symptoms in the different questionnaires.

### Factor analysis using truncated versions of PDI and ASRS / AQ

The factor analysis using a 3-factor model turned out not to show a satisfactory fit. This led us to perform another factor analysis using a 4-, and then a 5-factor model. Unlike the 4-factor model, the 5-factor one showed a satisfactory fit to the data, with an RMSEA of 0.027 and a CFI of 0.962. In the 5-factor model each factor contained items from only one questionnaire. The first factor (denoted *AQ factor*) had seven standardised loading estimates of at least 0.4, all items from AQ. Factors 2, 3, and 5 only comprised items from ASRS. While factor 2 included questions focusing on inattention (denoted *ASRS-Inattention factor*), factor 3 included hyperactivity questions (denoted *ASRS-Hyperactivity factor*) and factor 5 included impulsivity questions (denoted *ASRS-Impulsivity factor*). Finally, factor 4 had nine items of at least 0.4, all from PDI-21 (denoted *PDI factor*) (Table 4 and S5 Table). We performed a correlational analysis using the factors from the 5-factor model showing significant small correlations between the *PDI factor* and all the four other factors (Table 5 and S6 Table).

**Table 4:** 5-factor model (truncated questionnaires).

		Two-Tailed	
Factors	Items	Estimate	p-value
Factor 1 –AQ—social impairments	AQ20	0.689	0.000
	AQ22	0.700	0.000
	AQ26	0.683	0.000
	AQ35	0.447	0.000
	AQ36	0.562	0.000
	AQ42	0.519	0.000
	AQ45	0.784	0.000
Factor 2—ASRS—Inattention	ASRS2	0.630	0.000
	ASRS3	0.597	0.000
	ASRS4	0.784	0.000
	ASRS10	0.495	0.000
Factor 3 –ASRS—Hyperactivity	ASRS5	0.477	0.000
	ASRS6	0.769	0.000
	ASRS13	0.830	0.000
	ASRS14	0.408	0.000
Factor 4 PDI	PDINY02	0.455	0.000
	PDINY04	0.612	0.000
	PDINY05	0.552	0.000
	PDINY08	0.481	0.000
	PDINY09	0.669	0.000
	PDINY12	0.654	0.000
	PDINY18	0.493	0.000
	PDINY19	0.626	0.000
	PDINY20	0.582	0.000
Factor 5—ASRS—Impulsivity	ASRS16	0.471	0.000
	ASRS17	0.756	0.000
	ASRS18	0.558	0.000

List of items with standardised loadings of at least 0.4, on each of the five factors.

**Table 5:** Significant correlations between the PDI-factor and three other factors (from 5-factor model analysis on the truncated questionnaires).

		Two-Tailed	
		Estimate	p-value
Delusion-proneness with	AQ—social interaction and communication	0.212	0.000
	ASRS-Inattention	0.160	0.001
	ASRS-Hyperactivity	0.258	0.000
	ASRS-Impulsivity	0.235	0.000

## Discussion

In the present study we show that delusion-proneness correlates with both ASD- and ADHD-traits in a large sample of healthy individuals. The results suggested a weak, though significant, positive correlation between delusion-proneness scores and ASD trait scores, as well as a significant moderate positive correlation between delusion-proneness and ADHD traits. These observations remained unchanged when we studied a truncated version of the three questionnaires, only comprising items we had identified as the most prototypical to the disorder they characterised. We also performed a factor analysis on the truncated version of the questionnaires that supported the main results. A five-factor model that identified one factor that included PDI-21 items (PDI factor), another factor that included AQ-items (ASD factor) and three factors that included ASRS items (impulsivity factor, inattention factor, and hyperactivity factor). The analysis further showed small positive correlations that were highly significant, between the PDI factor and all the other factors. A similar, albeit somewhat less consistent, result was shown in a seven-factor model for the full version of the questionnaires.

Previously, research has focused on how different categorically defined mental disorders relate to each other, i.e. co-morbidity. Here, we have instead studied dimensions consisting of subclinical symptoms and focused on how these different traits relate to each other, i.e. trait co-morbidity. The analysis is motivated by the hypothesis that many psychiatric states are better described as dimensional rather than categorical disorders[31]. This has been suggested both for ASD[28] and ADHD[29,30]. For psychosis there seems to be a dimensional distribution of subclinical symptoms that is linked to a vulnerability to develop a manifest psychotic disorder[5]. It has been suggested that the dimensionality in subclinical symptoms is related to how effective information processing and control different individuals possess in various cognitive processes, i.e. cognitive core capacity[31]. The results of the present study support the hypothesis of cognitive core capacities (as there is a clustering of subclinical symptoms in highly specific factors) and that the cognitive core capacities for different processes relate to each other (as the factors correlate with each other). This implies that there may be both a degree of specificity related to the different traits but also a general overarching mechanism related to all traits—mirroring the idea that different psychiatric disorders are caused both by specific and general psychopathology factors[43,44].

In the present study we analysed a data set consisting of the full versions of questionnaires, and a data set comprising a truncated version of the questionnaires that was considered to be most prototypic for the different disorders (ASD, ADHD and psychosis). Analysing the full questionnaires led to the identification of seven factors that were in line with the different symptom ensembles we were expecting for each trait (hyperactivity factor, impulsivity factor, etc). The factor analysis revealed only one factor associated with delusion-proneness, which comprised most of the PDI items (fifteen out of twenty-one). Our results confirmed that delusion-proneness was positively associated with ADHD and ASD traits. Interestingly the delusion-proneness factor correlated with more factors representing ADHD-like symptoms than ASD. However, a potential limitation to these observations was the fact some items in the questionnaires targeted symptoms that were redundant across the three disorders studied here. Therefore the factors we obtained, and subsequent correlations might have been driven by shared non-prototypical symptoms and thereby confounded the results. This effect was minimized by having two psychiatrists rate how prototypical each statement/question was, and then only using the most specific items for further analyses. This more refined approach gave us similar results. Earlier studies have shown that attention deficits are common in both ADHD and schizophrenia[45] and that the incidence of ADHD was actually increased in populations at risk for psychosis[45]. Our present results extend these findings by showing a positive correlation between the severity of the ADHD trait and delusion-proneness in a non-clinical population. This was also the case when the ADHD symptoms were split into the main constituting factors (impulsivity, inattention, hyperactivity) as a part of both factor analyses. In other words, the more delusion-prone the individuals are, the greater the number of ADHD-like impulsivity, inattention and hyperactivity symptoms they display. It seems unlikely that the core basic mechanisms underlying attentional deficits are the same in ADHD and schizophrenia. Although the dopamine system seems to be central in both disorders, ADHD is thought to be caused by an hypo-dopaminergic system[46] while schizophrenia is considered related to both a subcortical hyper-dopaminergic system (hyper-stimulation of D2 receptors in subcortical regions) and a prefrontal hypo-dopaminergic system (deficit in dopamine transmission at D1 receptors in the prefrontal cortex)[47]. However, despite their seemingly different origin, the general results of these attentional deficits are similar: they interfere with normal input processing, leading to similar specific impairments.

As shown in previous studies, the presence of ADHD trait during childhood slightly increases the risk to develop psychosis later in life[23,48–50], and it has also been shown that the presence of ADHD symptoms in patients with schizophrenia was associated with more severe psychotic symptoms[51]. However, ADHD trait is not sufficient to predict the occurrence of a psychotic episode, as most patients suffering from ADHD in childhood will not develop psychosis during adulthood. This supports the idea that other systems are involved in the emergence of psychosis. In addition, as suggested by some previous studies[45,52], the kind of psychotic disorders co-occurring with certain ADHD symptoms might represent a specific variant/phenotype of psychosis. It is thus important not to consider ADHD trait as a core or ubiquitous symptom of psychosis. This brings support to the necessity to take ADHD tendencies into account in studies focusing on psychosis in general, in order to avoid drawing conclusions about underlying mechanisms supposedly related to psychosis, while they are in fact associated with hyperactivity or inattention.

### Limitations and conclusion

The results reported in the present study argue in favour of a more systematic evaluation of ASD- and ADHD-traits when specifically studying delusion-proneness in otherwise healthy individuals. Nevertheless, some limitations have to be taken into account when interpreting our results. First, though it was stressed twice in the recruitment process that participants had to be healthy and without any psychiatric history we cannot exclude the possibility that some of them had an ADHD or ASD diagnosis since they were recruited online without any more detailed clinical assessments. However, similar correlations were observed when only including individuals that scored below the conventional symptom thresholds on values in any of ASRS and AQ questionnaires. In addition, as we mentioned above, we only collected data from male individuals. It would be useful to run a similar study with both male and female individuals. Another limitation related to our sample is the fact we do not have information about the demographic and socio-economic background of the participants, which prevented us from fully characterising our sample population. Also, a large number of individuals scored high on our ADHD and ASD questionnaires suggesting that our sample did not mirror a general population. This limits the generalisation of our findings. However, the main results of this proof-of-concept study does not depend on demographic variables or on representing a normal population but on the relation between different subclinical symptoms—and given the large sample size it is well suited to show such relations.

It should be noted that in this study we are only demonstrating the existence of overlaps between those trait-related factors, and not making any inference in terms of causality, as correlations do not provide any information regarding causal relationship. Another limitation in our study, and common to most psychiatric research, is the fact our data is based on self-reported questionnaires. Indeed, the same psychiatric symptoms are reported by the same individual raises concerns about the reliability of the findings. In addition the wording used on the different items is also susceptible to interpretation biases. Some items can be interpreted in slightly different ways and might therefore fail to catch the aspects or dimensions for which they have been designed. One must thus be cautious when drawing conclusions, since part of the data might actually relate more to wording than characteristics of the trait *per se*.

Despite the limitations presented above, the present study supports the existence of subclinical co-morbidities between psychosis-proneness and ASD- or ADHD-traits, without drawing any conclusions in terms of causal relationship. Our results thus suggest that it is important to investigate these relationships in each individual study aiming at characterising delusion-proneness, as these relationships may be different depending on the sample. This also supports the need for more studies investigating in greater details those overlaps between psychosis-proneness, ADHD and ASD, as well as their underlying mechanisms.

## Supporting information

S1 Table Descriptive data with comparison to standard values (DOCX)

S2 Table List of selected items from each of the questionnaires (DOCX)

S3 Table 7-factor model (full questionnaires). Standardised loadings of all AQ-, ASRS- and PDI-items, on each of the 7 factors. Items with standardised loadings of at least 0.4 are reported in bold.
(DOCX)

S4 Table Correlations between the different factors from the 7-factor model analysis (full questionnaires). Significant correlations reported in bold
(DOCX)

S5 Table 5-factor model (truncated questionnaires). Standardised loadings of all the selected AQ-, ASRS- and PDI-items, on each of the 5 factors. Items with standardised loadings of at least 0.4 are reported in bold.
(DOCX)

S6 Table Significant correlations between the different factors from the 5-factor model analysis (DOCX)

S1 Appendix Correlation analyses. (DOCX)

S2 Appendix “Super-healthy” group. (DOCX)
