# Supplementary material for: Delusion-proneness displays comorbidity with traits of autistic-spectrum disorders and ADHD
Source: PLoS One. 2017 May 18;12(5):e0177820. doi: 10.1371/journal.pone.0177820 (PMC5436821; doi:10.1371/journal.pone.0177820)
Supplement: S1 Table — (DOCX) [file pone.0177820.s001.docx]

**Delusion-proneness displays comorbidity with traits of Autistic-Spectrum Disorders and ADHD**

**S1 Table. Descriptive data with comparison to standard values**

|  | Our sample | | Baron-Cohen *et al.*[1] | |
| --- | --- | --- | --- | --- |
|  | Mean (n=925) | SD | Mean (n=50) | SD |
| AQ | 15.79 | 6.352 | 20.2 | 4.8 |

|  | Our sample | | Oliveira *et al.*[2] | |
| --- | --- | --- | --- | --- |
|  | Mean (n=925) | SD | Mean (n=509) | SD |
| ASRS total | 29.78 | 10.355 | 28.42 | 9.14 |

|  | Our sample | | Peters *et al.*[3] | |
| --- | --- | --- | --- | --- |
|  | Mean (n=925) | SD | Mean | SD |
| PDI yes/no | 5.87 | 3.724 | 6.4 (n=187) | 4.4 |
| PDI distress | 13.95 | 11.702 | 14.4 (n=180) | 14.1 |
| PDI preoccupation | 15.4 | 12.084 | 15.1 (n=182) | 14.6 |
| PDI conviction | 17.91 | 13.016 | 20.1 (n=177) | 16.7 |

There were no missing data and the number of outliers was sufficiently small not to be considered of importance (between 9 to 13 outliers, depending of questionnaire - out of 925 participants).

**References**

1. Baron-Cohen S, Wheelwright S, Skinner R, Martin J, Clubley E. The Autism Spectrum Quotient : Evidence from Asperger syndrome/high functioning autism, males and females, scientists and mathematicians. J Autism Dev Disord. 2001;31: 5–17. doi:10.1023/A:1005653411471

2. Oliveira CT de, Hauck-Filho N, Dias ACG. College Adjustment as a Mediator Between Attention Deficit/Hyperactivity Disorder Symptoms and Work Self-Efficacy. Paid (Ribeirão Preto). 2016;26: 283–289. doi:10.1590/1982-43272665201607

3. Peters E, Joseph S, Day S, Garety P. Measuring Delusional Ideation: The 21-Item Peters et al. Delusions Inventor...: Joshua. Schizophr Bull. 2004;30: 1005–1022. doi:10.1037/t03329-000
