# Supplementary material for: Delusion-proneness displays comorbidity with traits of autistic-spectrum disorders and ADHD
Source: PLoS One. 2017 May 18;12(5):e0177820. doi: 10.1371/journal.pone.0177820 (PMC5436821; doi:10.1371/journal.pone.0177820)
Supplement: S2 Table — (DOCX) [file pone.0177820.s002.docx]

**Delusion-proneness displays comorbidity with traits of Autistic-Spectrum Disorders and ADHD**

**S2 Table.** **List of selected items from each of the questionnaires**

| Questionnaire | Selected items for each questionnaire |
| --- | --- |
| AQ | 1. I prefer to do things with others rather than on my own. |
|  | 2. I prefer to do things the same way over and over again. |
|  | 8. When I’m reading a story, I can easily imagine what the characters might look like. |
|  | 9. I am fascinated by dates. |
|  | 16. I tend to have very strong interests, which I get upset about if I can’t pursue. |
|  | 20. When I’m reading a story, I ﬁnd it difﬁcult to work out the characters’ intentions. |
|  | 22. I ﬁnd it hard to make new friends. |
|  | 25. It does not upset me if my daily routine is disturbed. |
|  | 26. I frequently ﬁnd that I don’t know how to keep a conversation going. |
|  | 35. I am often the last to understand the point of a joke. |
|  | 36. I ﬁnd it easy to work out what someone is thinking or feeling just by looking at their face. |
|  | 41. I like to collect information about categories of things (e.g. types of car, types of bird, types of train, types of plant, etc.). |
|  | 42. I ﬁnd it difﬁcult to imagine what it would be like to be someone else. |
|  | 45. I ﬁnd it difﬁcult to work out people’s intentions. |
|  | 2. How often do you have difficulty getting things in order when you have to do a task that requires organization? |
|  | 3. How often do you have problems remembering appointments or obligations? |
|  | 4. When you have a task that requires a lot of thought, how often do you avoid or delay getting started? |
|  | 5. How often do you fidget or squirm with your hands or feet when you have to sit down for a long time? |
|  | 6. How often do you feel overly active and compelled to do things, like you were driven by a motor? |
|  | 7. How often do you make careless mistakes when you have to work on a boring or difficult project? |
|  | 9. How often do you have difficulty concentrating on what people say to you, even when they are speaking to you directly? |
|  | 10. How often do you misplace or have difficulty finding things at home or at work? |
|  | 13. How often do you feel restless or fidgety? |
|  | 14. How often do you have difficulty unwinding and relaxing when you have time to yourself? |
|  | 16. When you’re in a conversation, how often do you find yourself finishing the sentences of the people you are talking to, before they can finish them themselves? |
|  | 17. How often do you have difficulty waiting your turn in situations when turn taking is required? |
|  | 18. How often do you interrupt others when they are busy? |
| PDI | 2. Do you ever feel as if things in magazines or on TV were written especially for you? |
|  | 4. Do you ever feel as if you are being persecuted in some way? |
|  | 5. Do you ever feel as if there is a conspiracy against you? |
|  | 8. Do you ever feel that you are especially close to God? |
|  | 9. Do you ever think people can communicate telepathically? |
|  | 12. Do you believe in the power of witchcraft, voodoo or the occult? |
|  | 18. Do your thoughts ever feel alien to you in some way? |
|  | 19. Have your thoughts ever been so vivid that you were worried other people would hear them? |
|  | 20. Do you ever feel as if your own thoughts were being echoed back to you? |
