# Supplementary material for: Delusion-proneness displays comorbidity with traits of autistic-spectrum disorders and ADHD
Source: PLoS One. 2017 May 18;12(5):e0177820. doi: 10.1371/journal.pone.0177820 (PMC5436821; doi:10.1371/journal.pone.0177820)
Supplement: S3 Table — Standardised loadings of all AQ-, ASRS- and PDI-items, on each of the 7 factors. Items with standardised loadings of at least 0.4 are reported in bold. (DOCX) [file pone.0177820.s003.docx]

**Delusion-proneness displays comorbidity with traits of Autistic-Spectrum Disorders and ADHD**

**S3 Table. 7-factor model (full questionnaires)**

|  | **Two-Tailed** | | | |
| --- | --- | --- | --- | --- |
| **Factor 1** | **Estimate** | **S.E.** | **Est./S.E.** | **p-value** |
| **AQ01** | **0.508** | **0.050** | **10.157** | **0.000** |
| AQ02 | 0.168 | 0.061 | 2.726 | 0.006 |
| AQ03 | -0.084 | 0.078 | -1.065 | 0.287 |
| AQ04 | 0.058 | 0.057 | 1.011 | 0.312 |
| AQ05 | 0.007 | 0.053 | 0.125 | 0.900 |
| AQ06 | -0.111 | 0.055 | -2.011 | 0.044 |
| AQ07 | 0.232 | 0.067 | 3.443 | 0.001 |
| AQ08 | -0.047 | 0.053 | -0.887 | 0.375 |
| AQ09 | -0.135 | 0.069 | -1.951 | 0.051 |
| AQ10 | 0.308 | 0.058 | 5.280 | 0.000 |
| **AQ11** | **0.821** | **0.048** | **17.201** | **0.000** |
| AQ12 | 0.063 | 0.061 | 1.042 | 0.297 |
| **AQ13** | **0.554** | **0.059** | **9.420** | **0.000** |
| AQ14 | 0.153 | 0.064 | 2.390 | 0.017 |
| **AQ15** | **0.609** | **0.047** | **13.027** | **0.000** |
| AQ16 | 0.080 | 0.058 | 1.395 | 0.163 |
| **AQ17** | **0.807** | **0.035** | **22.810** | **0.000** |
| AQ18 | -0.172 | 0.068 | -2.534 | 0.011 |
| AQ19 | -0.032 | 0.048 | -0.666 | 0.506 |
| AQ20 | 0.009 | 0.056 | 0.157 | 0.875 |
| AQ21 | 0.010 | 0.056 | 0.178 | 0.859 |
| **AQ22** | **0.666** | **0.050** | **13.384** | **0.000** |
| AQ23 | 0.074 | 0.054 | 1.378 | 0.168 |
| AQ24 | 0.156 | 0.056 | 2.809 | 0.005 |
| AQ25 | 0.200 | 0.059 | 3.391 | 0.001 |
| **AQ26** | **0.684** | **0.047** | **14.500** | **0.000** |
| AQ27 | -0.008 | 0.048 | -0.167 | 0.867 |
| AQ28 | 0.219 | 0.059 | 3.678 | 0.000 |
| AQ29 | -0.039 | 0.050 | -0.769 | 0.442 |
| AQ30 | -0.103 | 0.059 | -1.737 | 0.082 |
| AQ31 | 0.164 | 0.067 | 2.440 | 0.015 |
| AQ32 | 0.198 | 0.061 | 3.244 | 0.001 |
| AQ33 | 0.354 | 0.067 | 5.265 | 0.000 |
| **AQ34** | **0.516** | **0.068** | **7.613** | **0.000** |
| AQ35 | 0.125 | 0.070 | 1.785 | 0.074 |
| AQ36 | 0.032 | 0.056 | 0.567 | 0.571 |
| AQ37 | 0.276 | 0.061 | 4.502 | 0.000 |
| **AQ38** | **0.836** | **0.041** | **20.374** | **0.000** |
| AQ39 | 0.102 | 0.068 | 1.511 | 0.131 |
| AQ40 | 0.078 | 0.071 | 1.099 | 0.272 |
| AQ41 | -0.015 | 0.051 | -0.294 | 0.769 |
| AQ42 | 0.114 | 0.065 | 1.757 | 0.079 |
| AQ43 | 0.175 | 0.066 | 2.668 | 0.008 |
| **AQ44** | **0.910** | **0.046** | **19.799** | **0.000** |
| AQ45 | 0.097 | 0.069 | 1.392 | 0.164 |
| **AQ46** | **0.554** | **0.051** | **10.915** | **0.000** |
| **AQ47** | **0.917** | **0.039** | **23.514** | **0.000** |
| AQ48 | 0.064 | 0.067 | 0.951 | 0.342 |
| AQ49 | -0.102 | 0.061 | -1.690 | 0.091 |
| AQ50 | 0.174 | 0.068 | 2.566 | 0.010 |
| ASRS01 | 0.051 | 0.048 | 1.047 | 0.295 |
| ASRS02 | 0.063 | 0.058 | 1.092 | 0.275 |
| ASRS03 | 0.076 | 0.052 | 1.468 | 0.142 |
| ASRS04 | 0.151 | 0.064 | 2.371 | 0.018 |
| ASRS05 | -0.017 | 0.037 | -0.462 | 0.644 |
| ASRS06 | -0.087 | 0.042 | -2.103 | 0.035 |
| ASRS07 | -0.067 | 0.042 | -1.593 | 0.111 |
| ASRS08 | 0.012 | 0.038 | 0.307 | 0.759 |
| ASRS09 | 0.172 | 0.038 | 4.544 | 0.000 |
| ASRS10 | -0.040 | 0.044 | -0.909 | 0.363 |
| ASRS11 | 0.032 | 0.038 | 0.844 | 0.399 |
| ASRS12 | 0.023 | 0.041 | 0.569 | 0.569 |
| ASRS13 | -0.043 | 0.036 | -1.215 | 0.224 |
| ASRS14 | -0.005 | 0.037 | -0.150 | 0.881 |
| ASRS15 | -0.300 | 0.050 | -6.036 | 0.000 |
| ASRS16 | -0.010 | 0.034 | -0.292 | 0.770 |
| ASRS17 | -0.057 | 0.035 | -1.609 | 0.108 |
| ASRS18 | -0.042 | 0.039 | -1.092 | 0.275 |
| PDINY01 | 0.096 | 0.054 | 1.771 | 0.077 |
| PDINY02 | -0.031 | 0.070 | -0.447 | 0.655 |
| PDINY03 | -0.015 | 0.052 | -0.286 | 0.775 |
| PDINY04 | 0.091 | 0.067 | 1.368 | 0.171 |
| PDINY05 | 0.204 | 0.061 | 3.327 | 0.001 |
| PDINY06 | -0.134 | 0.059 | -2.278 | 0.023 |
| PDINY07 | 0.088 | 0.060 | 1.469 | 0.142 |
| PDINY08 | -0.188 | 0.087 | -2.154 | 0.031 |
| PDINY09 | 0.033 | 0.046 | 0.708 | 0.479 |
| PDINY10 | -0.143 | 0.058 | -2.457 | 0.014 |
| PDINY11 | -0.215 | 0.096 | -2.237 | 0.025 |
| PDINY12 | 0.047 | 0.055 | 0.841 | 0.400 |
| PDINY13 | 0.031 | 0.061 | 0.506 | 0.613 |
| PDINY14 | -0.077 | 0.055 | -1.396 | 0.163 |
| PDINY15 | 0.270 | 0.058 | 4.666 | 0.000 |
| PDINY16 | 0.111 | 0.060 | 1.851 | 0.064 |
| PDINY17 | 0.058 | 0.058 | 0.997 | 0.319 |
| PDINY18 | -0.068 | 0.053 | -1.298 | 0.194 |
| PDINY19 | 0.045 | 0.053 | 0.846 | 0.398 |
| PDINY20 | 0.052 | 0.053 | 0.979 | 0.328 |
| PDINY21 | -0.009 | 0.063 | -0.138 | 0.890 |
|  |  |  |  |  |
|  |  |  |  |  |
|  | **Two-Tailed** | | | |
| **Factor 2** | **Estimate** | **S.E.** | **Est./S.E.** | **p-value** |
| AQ01 | 0.087 | 0.055 | 1.582 | 0.114 |
| AQ02 | 0.065 | 0.054 | 1.217 | 0.224 |
| AQ03 | 0.020 | 0.055 | 0.368 | 0.713 |
| AQ04 | 0.141 | 0.054 | 2.626 | 0.009 |
| **AQ05** | **0.419** | **0.051** | **8.217** | **0.000** |
| **AQ06** | **0.536** | **0.045** | **11.919** | **0.000** |
| AQ07 | 0.143 | 0.058 | 2.480 | 0.013 |
| AQ08 | 0.118 | 0.068 | 1.732 | 0.083 |
| **AQ09** | **0.453** | **0.058** | **7.786** | **0.000** |
| AQ10 | -0.131 | 0.059 | -2.239 | 0.025 |
| AQ11 | 0.040 | 0.041 | 0.957 | 0.338 |
| **AQ12** | **0.619** | **0.057** | **10.896** | **0.000** |
| AQ13 | 0.002 | 0.051 | 0.039 | 0.969 |
| AQ14 | -0.096 | 0.056 | -1.709 | 0.088 |
| AQ15 | 0.119 | 0.051 | 2.343 | 0.019 |
| AQ16 | 0.125 | 0.052 | 2.397 | 0.017 |
| AQ17 | 0.028 | 0.042 | 0.660 | 0.509 |
| AQ18 | 0.008 | 0.037 | 0.205 | 0.838 |
| **AQ19** | **0.561** | **0.045** | **12.370** | **0.000** |
| AQ20 | -0.074 | 0.060 | -1.228 | 0.220 |
| AQ21 | 0.004 | 0.051 | 0.073 | 0.942 |
| AQ22 | -0.082 | 0.045 | -1.828 | 0.068 |
| **AQ23** | **0.578** | **0.048** | **12.020** | **0.000** |
| AQ24 | 0.086 | 0.053 | 1.628 | 0.104 |
| AQ25 | 0.051 | 0.053 | 0.960 | 0.337 |
| AQ26 | 0.012 | 0.039 | 0.316 | 0.752 |
| AQ27 | 0.116 | 0.068 | 1.715 | 0.086 |
| AQ28 | 0.289 | 0.051 | 5.692 | 0.000 |
| **AQ29** | **0.485** | **0.047** | **10.317** | **0.000** |
| AQ30 | 0.283 | 0.059 | 4.794 | 0.000 |
| AQ31 | 0.158 | 0.062 | 2.543 | 0.011 |
| AQ32 | -0.014 | 0.048 | -0.288 | 0.773 |
| AQ33 | -0.014 | 0.053 | -0.259 | 0.795 |
| AQ34 | 0.044 | 0.061 | 0.725 | 0.469 |
| AQ35 | -0.031 | 0.059 | -0.522 | 0.602 |
| AQ36 | -0.044 | 0.058 | -0.765 | 0.444 |
| AQ37 | 0.037 | 0.045 | 0.817 | 0.414 |
| AQ38 | -0.006 | 0.035 | -0.180 | 0.857 |
| AQ39 | 0.099 | 0.055 | 1.802 | 0.072 |
| AQ40 | 0.091 | 0.057 | 1.582 | 0.114 |
| AQ41 | 0.390 | 0.051 | 7.715 | 0.000 |
| AQ42 | -0.030 | 0.052 | -0.574 | 0.566 |
| AQ43 | 0.264 | 0.053 | 4.939 | 0.000 |
| AQ44 | -0.092 | 0.051 | -1.783 | 0.075 |
| AQ45 | -0.082 | 0.064 | -1.292 | 0.196 |
| AQ46 | -0.064 | 0.053 | -1.199 | 0.230 |
| AQ47 | -0.038 | 0.043 | -0.872 | 0.383 |
| AQ48 | -0.037 | 0.060 | -0.614 | 0.539 |
| AQ49 | 0.306 | 0.054 | 5.640 | 0.000 |
| AQ50 | 0.028 | 0.049 | 0.578 | 0.563 |
| ASRS01 | 0.005 | 0.039 | 0.130 | 0.897 |
| ASRS02 | -0.118 | 0.045 | -2.617 | 0.009 |
| ASRS03 | -0.077 | 0.042 | -1.827 | 0.068 |
| ASRS04 | -0.042 | 0.043 | -0.982 | 0.326 |
| ASRS05 | 0.004 | 0.034 | 0.131 | 0.896 |
| ASRS06 | 0.026 | 0.031 | 0.857 | 0.392 |
| ASRS07 | -0.132 | 0.043 | -3.087 | 0.002 |
| ASRS08 | -0.143 | 0.041 | -3.500 | 0.000 |
| ASRS09 | -0.051 | 0.038 | -1.344 | 0.179 |
| ASRS10 | -0.107 | 0.043 | -2.475 | 0.013 |
| ASRS11 | 0.107 | 0.041 | 2.608 | 0.009 |
| ASRS12 | -0.033 | 0.041 | -0.807 | 0.419 |
| ASRS13 | -0.060 | 0.035 | -1.742 | 0.081 |
| ASRS14 | 0.002 | 0.037 | 0.062 | 0.951 |
| ASRS15 | 0.003 | 0.031 | 0.097 | 0.923 |
| ASRS16 | 0.095 | 0.042 | 2.273 | 0.023 |
| ASRS17 | 0.007 | 0.031 | 0.240 | 0.810 |
| ASRS18 | -0.028 | 0.035 | -0.798 | 0.425 |
| PDINY01 | 0.177 | 0.057 | 3.087 | 0.002 |
| PDINY02 | 0.047 | 0.062 | 0.761 | 0.446 |
| PDINY03 | 0.187 | 0.056 | 3.331 | 0.001 |
| PDINY04 | -0.002 | 0.058 | -0.029 | 0.976 |
| PDINY05 | 0.158 | 0.063 | 2.494 | 0.013 |
| PDINY06 | 0.193 | 0.052 | 3.715 | 0.000 |
| PDINY07 | 0.305 | 0.058 | 5.282 | 0.000 |
| PDINY08 | -0.067 | 0.056 | -1.194 | 0.233 |
| PDINY09 | 0.053 | 0.053 | 1.007 | 0.314 |
| PDINY10 | 0.063 | 0.051 | 1.233 | 0.218 |
| PDINY11 | -0.065 | 0.062 | -1.047 | 0.295 |
| PDINY12 | -0.036 | 0.053 | -0.672 | 0.502 |
| PDINY13 | 0.095 | 0.058 | 1.641 | 0.101 |
| PDINY14 | 0.020 | 0.050 | 0.402 | 0.688 |
| PDINY15 | 0.109 | 0.056 | 1.948 | 0.051 |
| PDINY16 | -0.036 | 0.053 | -0.690 | 0.490 |
| PDINY17 | 0.038 | 0.052 | 0.738 | 0.460 |
| PDINY18 | 0.128 | 0.057 | 2.239 | 0.025 |
| PDINY19 | 0.140 | 0.061 | 2.276 | 0.023 |
| PDINY20 | 0.204 | 0.061 | 3.319 | 0.001 |
| PDINY21 | -0.095 | 0.063 | -1.520 | 0.128 |
|  |  |  |  |  |
|  |  |  |  |  |
|  | **Two-Tailed** | | | |
| **Factor 3** | **Estimate** | **S.E.** | **Est./S.E.** | **p-value** |
| AQ01 | -0.028 | 0.052 | -0.539 | 0.590 |
| AQ02 | 0.127 | 0.058 | 2.206 | 0.027 |
| AQ03 | -0.079 | 0.076 | -1.044 | 0.297 |
| AQ04 | 0.271 | 0.055 | 4.923 | 0.000 |
| AQ05 | -0.013 | 0.049 | -0.260 | 0.795 |
| AQ06 | -0.056 | 0.054 | -1.034 | 0.301 |
| **AQ07** | **0.462** | **0.052** | **8.872** | **0.000** |
| AQ08 | -0.294 | 0.075 | -3.908 | 0.000 |
| AQ09 | 0.112 | 0.064 | 1.744 | 0.081 |
| AQ10 | 0.006 | 0.050 | 0.124 | 0.901 |
| AQ11 | -0.051 | 0.043 | -1.182 | 0.237 |
| AQ12 | 0.017 | 0.051 | 0.337 | 0.736 |
| AQ13 | 0.076 | 0.058 | 1.307 | 0.191 |
| AQ14 | -0.398 | 0.055 | -7.200 | 0.000 |
| AQ15 | -0.013 | 0.050 | -0.264 | 0.792 |
| AQ16 | 0.372 | 0.050 | 7.361 | 0.000 |
| AQ17 | 0.038 | 0.045 | 0.845 | 0.398 |
| **AQ18** | **0.753** | **0.041** | **18.425** | **0.000** |
| AQ19 | -0.053 | 0.048 | -1.107 | 0.268 |
| AQ20 | 0.085 | 0.076 | 1.110 | 0.267 |
| AQ21 | -0.054 | 0.057 | -0.935 | 0.350 |
| AQ22 | -0.056 | 0.045 | -1.261 | 0.207 |
| AQ23 | 0.034 | 0.048 | 0.706 | 0.480 |
| AQ24 | 0.094 | 0.054 | 1.744 | 0.081 |
| AQ25 | 0.180 | 0.057 | 3.175 | 0.001 |
| AQ26 | -0.027 | 0.039 | -0.692 | 0.489 |
| AQ27 | 0.015 | 0.044 | 0.345 | 0.730 |
| AQ28 | 0.168 | 0.056 | 3.011 | 0.003 |
| AQ29 | 0.005 | 0.048 | 0.111 | 0.911 |
| AQ30 | -0.011 | 0.048 | -0.234 | 0.815 |
| AQ31 | -0.005 | 0.055 | -0.098 | 0.922 |
| AQ32 | 0.114 | 0.057 | 2.003 | 0.045 |
| AQ33 | 0.316 | 0.058 | 5.441 | 0.000 |
| AQ34 | -0.055 | 0.066 | -0.834 | 0.405 |
| AQ35 | 0.159 | 0.069 | 2.315 | 0.021 |
| AQ36 | -0.189 | 0.068 | -2.768 | 0.006 |
| AQ37 | 0.146 | 0.055 | 2.653 | 0.008 |
| AQ38 | -0.078 | 0.043 | -1.811 | 0.070 |
| **AQ39** | **0.510** | **0.056** | **9.126** | **0.000** |
| AQ40 | -0.201 | 0.076 | -2.659 | 0.008 |
| AQ41 | 0.110 | 0.057 | 1.925 | 0.054 |
| AQ42 | 0.143 | 0.061 | 2.351 | 0.019 |
| AQ43 | 0.082 | 0.058 | 1.403 | 0.161 |
| AQ44 | 0.139 | 0.055 | 2.514 | 0.012 |
| AQ45 | 0.051 | 0.057 | 0.899 | 0.369 |
| AQ46 | 0.069 | 0.050 | 1.378 | 0.168 |
| AQ47 | 0.012 | 0.041 | 0.286 | 0.775 |
| AQ48 | 0.290 | 0.069 | 4.205 | 0.000 |
| AQ49 | -0.173 | 0.057 | -3.047 | 0.002 |
| AQ50 | -0.133 | 0.068 | -1.949 | 0.051 |
| ASRS01 | 0.133 | 0.041 | 3.288 | 0.001 |
| ASRS02 | 0.029 | 0.038 | 0.768 | 0.442 |
| ASRS03 | 0.221 | 0.045 | 4.954 | 0.000 |
| ASRS04 | 0.034 | 0.038 | 0.884 | 0.377 |
| ASRS05 | -0.014 | 0.035 | -0.389 | 0.697 |
| ASRS06 | 0.073 | 0.049 | 1.490 | 0.136 |
| ASRS07 | 0.018 | 0.034 | 0.521 | 0.603 |
| ASRS08 | 0.027 | 0.033 | 0.831 | 0.406 |
| ASRS09 | 0.097 | 0.039 | 2.500 | 0.012 |
| ASRS10 | 0.127 | 0.040 | 3.197 | 0.001 |
| ASRS11 | 0.085 | 0.040 | 2.130 | 0.033 |
| ASRS12 | 0.362 | 0.047 | 7.720 | 0.000 |
| ASRS13 | 0.052 | 0.046 | 1.128 | 0.259 |
| ASRS14 | 0.120 | 0.048 | 2.516 | 0.012 |
| **ASRS15** | **0.568** | **0.034** | **16.903** | **0.000** |
| **ASRS16** | **0.405** | **0.039** | **10.424** | **0.000** |
| **ASRS17** | **0.570** | **0.040** | **14.339** | **0.000** |
| **ASRS18** | **0.479** | **0.039** | **12.129** | **0.000** |
| PDINY01 | 0.042 | 0.049 | 0.855 | 0.393 |
| PDINY02 | 0.092 | 0.071 | 1.283 | 0.199 |
| PDINY03 | 0.017 | 0.052 | 0.325 | 0.745 |
| PDINY04 | -0.005 | 0.056 | -0.094 | 0.925 |
| PDINY05 | -0.022 | 0.047 | -0.471 | 0.637 |
| PDINY06 | 0.110 | 0.053 | 2.091 | 0.037 |
| PDINY07 | 0.110 | 0.057 | 1.929 | 0.054 |
| PDINY08 | -0.013 | 0.043 | -0.301 | 0.763 |
| PDINY09 | -0.068 | 0.051 | -1.328 | 0.184 |
| PDINY10 | -0.088 | 0.053 | -1.669 | 0.095 |
| PDINY11 | 0.065 | 0.053 | 1.229 | 0.219 |
| PDINY12 | -0.054 | 0.055 | -0.968 | 0.333 |
| PDINY13 | 0.042 | 0.058 | 0.731 | 0.465 |
| PDINY14 | 0.093 | 0.053 | 1.764 | 0.078 |
| PDINY15 | 0.006 | 0.045 | 0.125 | 0.901 |
| PDINY16 | -0.049 | 0.054 | -0.908 | 0.364 |
| PDINY17 | 0.029 | 0.055 | 0.522 | 0.602 |
| PDINY18 | -0.022 | 0.048 | -0.449 | 0.653 |
| PDINY19 | -0.021 | 0.054 | -0.384 | 0.701 |
| PDINY20 | -0.047 | 0.053 | -0.884 | 0.377 |
| PDINY21 | -0.069 | 0.063 | -1.088 | 0.277 |
|  |  |  |  |  |
|  |  |  |  |  |
|  | **Two-Tailed** | | | |
| **Factor 4** | **Estimate** | **S.E.** | **Est./S.E.** | **p-value** |
| AQ01 | -0.106 | 0.067 | -1.586 | 0.113 |
| AQ02 | 0.119 | 0.060 | 1.965 | 0.049 |
| **AQ03** | **0.418** | **0.083** | **5.050** | **0.000** |
| AQ04 | 0.044 | 0.062 | 0.711 | 0.477 |
| AQ05 | -0.121 | 0.065 | -1.866 | 0.062 |
| AQ06 | -0.013 | 0.051 | -0.262 | 0.794 |
| AQ07 | 0.153 | 0.069 | 2.217 | 0.027 |
| **AQ08** | **0.544** | **0.076** | **7.164** | **0.000** |
| AQ09 | 0.300 | 0.072 | 4.155 | 0.000 |
| AQ10 | 0.369 | 0.066 | 5.575 | 0.000 |
| AQ11 | 0.177 | 0.095 | 1.869 | 0.062 |
| AQ12 | -0.178 | 0.089 | -2.009 | 0.044 |
| AQ13 | -0.023 | 0.058 | -0.406 | 0.684 |
| AQ14 | 0.287 | 0.065 | 4.395 | 0.000 |
| AQ15 | 0.095 | 0.073 | 1.302 | 0.193 |
| AQ16 | 0.079 | 0.060 | 1.323 | 0.186 |
| AQ17 | -0.096 | 0.071 | -1.349 | 0.177 |
| AQ18 | 0.002 | 0.049 | 0.049 | 0.961 |
| AQ19 | 0.190 | 0.070 | 2.723 | 0.006 |
| **AQ20** | **0.624** | **0.061** | **10.255** | **0.000** |
| AQ21 | 0.214 | 0.058 | 3.694 | 0.000 |
| AQ22 | 0.229 | 0.069 | 3.312 | 0.001 |
| AQ23 | -0.087 | 0.079 | -1.092 | 0.275 |
| AQ24 | 0.098 | 0.063 | 1.561 | 0.119 |
| AQ25 | 0.063 | 0.063 | 0.998 | 0.318 |
| AQ26 | 0.223 | 0.074 | 2.993 | 0.003 |
| **AQ27** | **0.644** | **0.058** | **11.106** | **0.000** |
| AQ28 | 0.046 | 0.062 | 0.744 | 0.457 |
| AQ29 | 0.019 | 0.054 | 0.345 | 0.730 |
| AQ30 | -0.344 | 0.060 | -5.702 | 0.000 |
| **AQ31** | **0.410** | **0.079** | **5.185** | **0.000** |
| AQ32 | 0.264 | 0.070 | 3.770 | 0.000 |
| AQ33 | 0.167 | 0.076 | 2.194 | 0.028 |
| AQ34 | 0.203 | 0.076 | 2.670 | 0.008 |
| AQ35 | 0.348 | 0.067 | 5.164 | 0.000 |
| **AQ36** | **0.635** | **0.054** | **11.734** | **0.000** |
| AQ37 | 0.220 | 0.077 | 2.847 | 0.004 |
| AQ38 | 0.081 | 0.083 | 0.983 | 0.326 |
| AQ39 | 0.239 | 0.069 | 3.484 | 0.000 |
| AQ40 | 0.374 | 0.065 | 5.734 | 0.000 |
| AQ41 | 0.172 | 0.064 | 2.693 | 0.007 |
| AQ42 | 0.396 | 0.055 | 7.235 | 0.000 |
| AQ43 | 0.108 | 0.066 | 1.638 | 0.101 |
| AQ44 | -0.045 | 0.045 | -0.988 | 0.323 |
| **AQ45** | **0.716** | **0.053** | **13.452** | **0.000** |
| AQ46 | 0.127 | 0.070 | 1.812 | 0.070 |
| AQ47 | -0.041 | 0.054 | -0.761 | 0.447 |
| AQ48 | 0.333 | 0.066 | 5.003 | 0.000 |
| AQ49 | 0.010 | 0.051 | 0.192 | 0.848 |
| AQ50 | 0.369 | 0.062 | 5.960 | 0.000 |
| ASRS01 | 0.083 | 0.061 | 1.352 | 0.176 |
| ASRS02 | 0.093 | 0.064 | 1.452 | 0.147 |
| ASRS03 | -0.021 | 0.043 | -0.501 | 0.616 |
| ASRS04 | -0.030 | 0.056 | -0.529 | 0.597 |
| ASRS05 | -0.026 | 0.040 | -0.655 | 0.513 |
| ASRS06 | -0.012 | 0.036 | -0.335 | 0.737 |
| ASRS07 | 0.011 | 0.040 | 0.267 | 0.789 |
| ASRS08 | -0.013 | 0.036 | -0.368 | 0.713 |
| ASRS09 | 0.067 | 0.042 | 1.582 | 0.114 |
| ASRS10 | 0.002 | 0.042 | 0.059 | 0.953 |
| ASRS11 | 0.086 | 0.048 | 1.803 | 0.071 |
| ASRS12 | 0.035 | 0.047 | 0.731 | 0.465 |
| ASRS13 | -0.005 | 0.033 | -0.143 | 0.886 |
| ASRS14 | 0.085 | 0.042 | 2.027 | 0.043 |
| ASRS15 | -0.078 | 0.046 | -1.706 | 0.088 |
| ASRS16 | -0.157 | 0.047 | -3.385 | 0.001 |
| ASRS17 | -0.024 | 0.039 | -0.615 | 0.539 |
| ASRS18 | 0.000 | 0.038 | -0.012 | 0.991 |
| PDINY01 | -0.085 | 0.053 | -1.609 | 0.108 |
| PDINY02 | 0.074 | 0.082 | 0.900 | 0.368 |
| PDINY03 | -0.057 | 0.057 | -0.998 | 0.318 |
| PDINY04 | 0.032 | 0.061 | 0.531 | 0.595 |
| PDINY05 | -0.002 | 0.051 | -0.041 | 0.967 |
| PDINY06 | -0.045 | 0.060 | -0.763 | 0.445 |
| PDINY07 | -0.028 | 0.054 | -0.529 | 0.597 |
| PDINY08 | 0.199 | 0.111 | 1.796 | 0.072 |
| PDINY09 | -0.174 | 0.059 | -2.942 | 0.003 |
| PDINY10 | 0.143 | 0.062 | 2.294 | 0.022 |
| PDINY11 | 0.232 | 0.120 | 1.928 | 0.054 |
| PDINY12 | -0.110 | 0.081 | -1.356 | 0.175 |
| PDINY13 | -0.069 | 0.063 | -1.099 | 0.272 |
| PDINY14 | -0.055 | 0.055 | -1.002 | 0.317 |
| PDINY15 | -0.031 | 0.051 | -0.613 | 0.540 |
| PDINY16 | -0.061 | 0.059 | -1.039 | 0.299 |
| PDINY17 | -0.038 | 0.061 | -0.629 | 0.529 |
| PDINY18 | -0.026 | 0.054 | -0.477 | 0.634 |
| PDINY19 | 0.028 | 0.053 | 0.537 | 0.591 |
| PDINY20 | -0.119 | 0.067 | -1.783 | 0.075 |
| PDINY21 | 0.094 | 0.079 | 1.196 | 0.232 |
|  |  |  |  |  |
|  |  |  |  |  |
|  | **Two-Tailed** | | | |
| **Factor 5** | **Estimate** | **S.E.** | **Est./S.E.** | **p-value** |
| AQ01 | 0.015 | 0.051 | 0.285 | 0.776 |
| AQ02 | -0.107 | 0.051 | -2.083 | 0.037 |
| AQ03 | -0.060 | 0.066 | -0.901 | 0.368 |
| AQ04 | 0.052 | 0.052 | 0.995 | 0.320 |
| AQ05 | 0.084 | 0.055 | 1.530 | 0.126 |
| AQ06 | 0.035 | 0.047 | 0.755 | 0.450 |
| AQ07 | 0.150 | 0.060 | 2.484 | 0.013 |
| AQ08 | -0.087 | 0.069 | -1.263 | 0.206 |
| AQ09 | 0.176 | 0.067 | 2.634 | 0.008 |
| AQ10 | -0.020 | 0.049 | -0.396 | 0.692 |
| AQ11 | -0.033 | 0.046 | -0.730 | 0.465 |
| AQ12 | 0.022 | 0.047 | 0.470 | 0.638 |
| AQ13 | 0.206 | 0.056 | 3.666 | 0.000 |
| AQ14 | -0.100 | 0.054 | -1.845 | 0.065 |
| AQ15 | -0.103 | 0.048 | -2.158 | 0.031 |
| AQ16 | 0.146 | 0.053 | 2.734 | 0.006 |
| AQ17 | -0.072 | 0.045 | -1.600 | 0.110 |
| AQ18 | -0.043 | 0.045 | -0.963 | 0.336 |
| AQ19 | -0.001 | 0.045 | -0.021 | 0.983 |
| AQ20 | 0.135 | 0.078 | 1.748 | 0.081 |
| AQ21 | 0.120 | 0.055 | 2.169 | 0.030 |
| AQ22 | 0.092 | 0.050 | 1.840 | 0.066 |
| AQ23 | 0.090 | 0.053 | 1.689 | 0.091 |
| AQ24 | -0.072 | 0.053 | -1.362 | 0.173 |
| AQ25 | 0.005 | 0.053 | 0.089 | 0.929 |
| AQ26 | 0.165 | 0.048 | 3.422 | 0.001 |
| AQ27 | 0.013 | 0.048 | 0.266 | 0.791 |
| AQ28 | -0.057 | 0.052 | -1.092 | 0.275 |
| AQ29 | -0.204 | 0.052 | -3.908 | 0.000 |
| AQ30 | -0.033 | 0.051 | -0.641 | 0.521 |
| AQ31 | -0.043 | 0.056 | -0.776 | 0.438 |
| AQ32 | -0.041 | 0.050 | -0.826 | 0.409 |
| AQ33 | 0.082 | 0.063 | 1.303 | 0.192 |
| AQ34 | -0.097 | 0.067 | -1.457 | 0.145 |
| AQ35 | 0.216 | 0.070 | 3.085 | 0.002 |
| AQ36 | -0.009 | 0.047 | -0.186 | 0.852 |
| AQ37 | 0.009 | 0.046 | 0.200 | 0.841 |
| AQ38 | 0.028 | 0.044 | 0.636 | 0.525 |
| AQ39 | 0.043 | 0.053 | 0.815 | 0.415 |
| AQ40 | -0.115 | 0.064 | -1.785 | 0.074 |
| AQ41 | 0.081 | 0.055 | 1.460 | 0.144 |
| AQ42 | 0.075 | 0.057 | 1.311 | 0.190 |
| AQ43 | -0.076 | 0.052 | -1.482 | 0.138 |
| AQ44 | 0.008 | 0.036 | 0.207 | 0.836 |
| AQ45 | 0.094 | 0.062 | 1.520 | 0.128 |
| AQ46 | 0.028 | 0.051 | 0.540 | 0.589 |
| AQ47 | -0.011 | 0.040 | -0.283 | 0.777 |
| AQ48 | -0.042 | 0.061 | -0.698 | 0.485 |
| AQ49 | -0.024 | 0.049 | -0.483 | 0.629 |
| AQ50 | -0.051 | 0.055 | -0.924 | 0.355 |
| ASRS01 | -0.058 | 0.037 | -1.551 | 0.121 |
| ASRS02 | -0.029 | 0.036 | -0.798 | 0.425 |
| ASRS03 | 0.058 | 0.044 | 1.325 | 0.185 |
| ASRS04 | -0.036 | 0.038 | -0.938 | 0.348 |
| ASRS05 | 0.043 | 0.038 | 1.112 | 0.266 |
| ASRS06 | 0.064 | 0.036 | 1.781 | 0.075 |
| ASRS07 | 0.048 | 0.040 | 1.203 | 0.229 |
| ASRS08 | 0.055 | 0.040 | 1.397 | 0.162 |
| ASRS09 | 0.104 | 0.038 | 2.715 | 0.007 |
| ASRS10 | 0.073 | 0.040 | 1.835 | 0.067 |
| ASRS11 | 0.072 | 0.041 | 1.775 | 0.076 |
| ASRS12 | 0.033 | 0.042 | 0.795 | 0.427 |
| ASRS13 | 0.033 | 0.033 | 0.985 | 0.325 |
| ASRS14 | -0.005 | 0.038 | -0.120 | 0.904 |
| ASRS15 | -0.075 | 0.040 | -1.850 | 0.064 |
| ASRS16 | -0.017 | 0.036 | -0.487 | 0.627 |
| ASRS17 | -0.061 | 0.036 | -1.669 | 0.095 |
| ASRS18 | -0.106 | 0.041 | -2.608 | 0.009 |
| PDINY01 | 0.357 | 0.051 | 7.039 | 0.000 |
| **PDINY02** | **0.472** | **0.062** | **7.581** | **0.000** |
| PDINY03 | 0.397 | 0.050 | 7.876 | 0.000 |
| **PDINY04** | **0.557** | **0.053** | **10.434** | **0.000** |
| **PDINY05** | **0.469** | **0.057** | **8.198** | **0.000** |
| **PDINY06** | **0.567** | **0.044** | **12.952** | **0.000** |
| **PDINY07** | **0.437** | **0.051** | **8.652** | **0.000** |
| **PDINY08** | **0.740** | **0.052** | **14.213** | **0.000** |
| **PDINY09** | **0.541** | **0.047** | **11.530** | **0.000** |
| PDINY10 | 0.344 | 0.053 | 6.500 | 0.000 |
| **PDINY11** | **0.778** | **0.053** | **14.639** | **0.000** |
| **PDINY12** | **0.614** | **0.049** | **12.505** | **0.000** |
| PDINY13 | 0.216 | 0.062 | 3.455 | 0.001 |
| PDINY14 | 0.280 | 0.053 | 5.296 | 0.000 |
| **PDINY15** | **0.418** | **0.048** | **8.663** | **0.000** |
| PDINY16 | 0.282 | 0.056 | 5.042 | 0.000 |
| **PDINY17** | **0.521** | **0.047** | **11.177** | **0.000** |
| **PDINY18** | **0.419** | **0.054** | **7.781** | **0.000** |
| **PDINY19** | **0.549** | **0.053** | **10.324** | **0.000** |
| **PDINY20** | **0.488** | **0.054** | **9.014** | **0.000** |
| **PDINY21** | **0.463** | **0.065** | **7.166** | **0.000** |
|  |  |  |  |  |
|  |  |  |  |  |
|  | **Two-Tailed** | | | |
| **Factor 6** | **Estimate** | **S.E.** | **Est./S.E.** | **p-value** |
| AQ01 | -0.079 | 0.064 | -1.239 | 0.215 |
| AQ02 | -0.197 | 0.054 | -3.640 | 0.000 |
| AQ03 | 0.323 | 0.086 | 3.742 | 0.000 |
| AQ04 | 0.235 | 0.052 | 4.510 | 0.000 |
| AQ05 | 0.055 | 0.052 | 1.050 | 0.294 |
| AQ06 | -0.052 | 0.048 | -1.078 | 0.281 |
| AQ07 | -0.039 | 0.043 | -0.899 | 0.369 |
| AQ08 | 0.284 | 0.097 | 2.932 | 0.003 |
| AQ09 | -0.073 | 0.063 | -1.157 | 0.247 |
| AQ10 | 0.156 | 0.068 | 2.313 | 0.021 |
| AQ11 | 0.149 | 0.087 | 1.717 | 0.086 |
| AQ12 | -0.022 | 0.053 | -0.423 | 0.672 |
| AQ13 | -0.243 | 0.069 | -3.505 | 0.000 |
| AQ14 | -0.062 | 0.064 | -0.979 | 0.328 |
| AQ15 | 0.007 | 0.062 | 0.115 | 0.908 |
| AQ16 | 0.022 | 0.049 | 0.446 | 0.656 |
| AQ17 | -0.093 | 0.083 | -1.128 | 0.259 |
| AQ18 | -0.018 | 0.042 | -0.427 | 0.670 |
| AQ19 | -0.019 | 0.051 | -0.381 | 0.703 |
| AQ20 | -0.036 | 0.058 | -0.614 | 0.539 |
| AQ21 | -0.136 | 0.056 | -2.436 | 0.015 |
| AQ22 | 0.036 | 0.056 | 0.638 | 0.523 |
| AQ23 | 0.096 | 0.058 | 1.660 | 0.097 |
| AQ24 | 0.010 | 0.054 | 0.192 | 0.848 |
| AQ25 | -0.104 | 0.056 | -1.864 | 0.062 |
| AQ26 | 0.101 | 0.067 | 1.503 | 0.133 |
| AQ27 | 0.199 | 0.092 | 2.151 | 0.031 |
| AQ28 | 0.071 | 0.060 | 1.189 | 0.234 |
| AQ29 | -0.046 | 0.053 | -0.871 | 0.384 |
| AQ30 | -0.086 | 0.059 | -1.463 | 0.144 |
| AQ31 | 0.164 | 0.082 | 2.016 | 0.044 |
| AQ32 | 0.259 | 0.060 | 4.299 | 0.000 |
| AQ33 | 0.079 | 0.055 | 1.431 | 0.152 |
| AQ34 | -0.259 | 0.085 | -3.054 | 0.002 |
| AQ35 | -0.012 | 0.053 | -0.218 | 0.827 |
| AQ36 | 0.120 | 0.090 | 1.333 | 0.183 |
| AQ37 | 0.344 | 0.063 | 5.441 | 0.000 |
| AQ38 | 0.123 | 0.082 | 1.502 | 0.133 |
| AQ39 | -0.017 | 0.045 | -0.386 | 0.700 |
| AQ40 | -0.194 | 0.089 | -2.178 | 0.029 |
| AQ41 | -0.053 | 0.052 | -1.025 | 0.306 |
| AQ42 | -0.089 | 0.065 | -1.371 | 0.170 |
| AQ43 | -0.326 | 0.056 | -5.794 | 0.000 |
| AQ44 | -0.385 | 0.094 | -4.099 | 0.000 |
| AQ45 | 0.082 | 0.086 | 0.958 | 0.338 |
| AQ46 | 0.049 | 0.062 | 0.787 | 0.431 |
| AQ47 | -0.282 | 0.092 | -3.071 | 0.002 |
| AQ48 | -0.116 | 0.074 | -1.560 | 0.119 |
| AQ49 | -0.214 | 0.060 | -3.572 | 0.000 |
| AQ50 | -0.091 | 0.076 | -1.200 | 0.230 |
| **ASRS01** | **0.503** | **0.037** | **13.657** | **0.000** |
| **ASRS02** | **0.637** | **0.036** | **17.900** | **0.000** |
| **ASRS03** | **0.474** | **0.041** | **11.701** | **0.000** |
| **ASRS04** | **0.629** | **0.043** | **14.721** | **0.000** |
| ASRS05 | 0.243 | 0.047 | 5.130 | 0.000 |
| ASRS06 | -0.025 | 0.042 | -0.587 | 0.557 |
| ASRS07 | 0.352 | 0.041 | 8.572 | 0.000 |
| **ASRS08** | **0.411** | **0.042** | **9.809** | **0.000** |
| ASRS09 | 0.230 | 0.047 | 4.872 | 0.000 |
| **ASRS10** | **0.437** | **0.039** | **11.172** | **0.000** |
| ASRS11 | 0.311 | 0.044 | 7.143 | 0.000 |
| ASRS12 | 0.169 | 0.043 | 3.947 | 0.000 |
| ASRS13 | 0.011 | 0.043 | 0.261 | 0.794 |
| ASRS14 | 0.086 | 0.044 | 1.964 | 0.050 |
| ASRS15 | 0.135 | 0.049 | 2.784 | 0.005 |
| ASRS16 | -0.014 | 0.039 | -0.354 | 0.723 |
| ASRS17 | 0.019 | 0.035 | 0.531 | 0.596 |
| ASRS18 | 0.116 | 0.043 | 2.695 | 0.007 |
| PDINY01 | 0.146 | 0.053 | 2.785 | 0.005 |
| PDINY02 | -0.248 | 0.068 | -3.660 | 0.000 |
| PDINY03 | 0.031 | 0.048 | 0.637 | 0.524 |
| PDINY04 | -0.055 | 0.054 | -1.024 | 0.306 |
| PDINY05 | 0.078 | 0.055 | 1.407 | 0.159 |
| PDINY06 | 0.002 | 0.047 | 0.033 | 0.974 |
| PDINY07 | 0.094 | 0.051 | 1.853 | 0.064 |
| PDINY08 | -0.251 | 0.088 | -2.838 | 0.005 |
| PDINY09 | 0.042 | 0.051 | 0.822 | 0.411 |
| PDINY10 | -0.008 | 0.050 | -0.152 | 0.879 |
| PDINY11 | -0.209 | 0.098 | -2.127 | 0.033 |
| PDINY12 | -0.133 | 0.064 | -2.096 | 0.036 |
| PDINY13 | 0.130 | 0.059 | 2.227 | 0.026 |
| PDINY14 | 0.131 | 0.055 | 2.373 | 0.018 |
| PDINY15 | 0.162 | 0.056 | 2.888 | 0.004 |
| PDINY16 | 0.143 | 0.058 | 2.473 | 0.013 |
| PDINY17 | 0.032 | 0.052 | 0.615 | 0.539 |
| PDINY18 | 0.158 | 0.053 | 2.971 | 0.003 |
| PDINY19 | 0.003 | 0.046 | 0.061 | 0.951 |
| PDINY20 | 0.035 | 0.054 | 0.645 | 0.519 |
| PDINY21 | 0.083 | 0.071 | 1.160 | 0.246 |
|  |  |  |  |  |
|  |  |  |  |  |
|  | **Two-Tailed** | | | |
| **Factor 7** | **Estimate** | **S.E.** | **Est./S.E.** | **p-value** |
| AQ01 | -0.017 | 0.047 | -0.369 | 0.712 |
| AQ02 | 0.057 | 0.050 | 1.132 | 0.258 |
| AQ03 | -0.002 | 0.056 | -0.027 | 0.978 |
| AQ04 | -0.022 | 0.049 | -0.443 | 0.658 |
| AQ05 | 0.050 | 0.052 | 0.969 | 0.333 |
| AQ06 | 0.074 | 0.049 | 1.516 | 0.130 |
| AQ07 | -0.008 | 0.040 | -0.199 | 0.842 |
| AQ08 | 0.084 | 0.061 | 1.384 | 0.166 |
| AQ09 | -0.076 | 0.053 | -1.436 | 0.151 |
| AQ10 | -0.071 | 0.052 | -1.370 | 0.171 |
| AQ11 | -0.088 | 0.050 | -1.754 | 0.079 |
| AQ12 | 0.134 | 0.065 | 2.050 | 0.040 |
| AQ13 | 0.012 | 0.046 | 0.253 | 0.800 |
| AQ14 | 0.101 | 0.053 | 1.923 | 0.055 |
| AQ15 | 0.018 | 0.045 | 0.405 | 0.685 |
| AQ16 | 0.034 | 0.046 | 0.738 | 0.460 |
| AQ17 | -0.020 | 0.038 | -0.530 | 0.596 |
| AQ18 | 0.000 | 0.043 | 0.007 | 0.995 |
| AQ19 | -0.055 | 0.045 | -1.212 | 0.226 |
| AQ20 | 0.101 | 0.066 | 1.536 | 0.125 |
| AQ21 | 0.164 | 0.053 | 3.079 | 0.002 |
| AQ22 | -0.102 | 0.050 | -2.048 | 0.041 |
| AQ23 | 0.018 | 0.044 | 0.412 | 0.681 |
| AQ24 | -0.055 | 0.049 | -1.103 | 0.270 |
| AQ25 | 0.124 | 0.052 | 2.376 | 0.017 |
| AQ26 | -0.075 | 0.046 | -1.634 | 0.102 |
| AQ27 | -0.137 | 0.057 | -2.402 | 0.016 |
| AQ28 | -0.040 | 0.049 | -0.819 | 0.413 |
| AQ29 | -0.036 | 0.045 | -0.802 | 0.422 |
| AQ30 | 0.023 | 0.050 | 0.469 | 0.639 |
| AQ31 | -0.028 | 0.055 | -0.516 | 0.606 |
| AQ32 | -0.098 | 0.051 | -1.936 | 0.053 |
| AQ33 | -0.006 | 0.052 | -0.108 | 0.914 |
| AQ34 | 0.048 | 0.055 | 0.878 | 0.380 |
| AQ35 | 0.059 | 0.064 | 0.914 | 0.361 |
| AQ36 | 0.027 | 0.050 | 0.536 | 0.592 |
| AQ37 | 0.016 | 0.044 | 0.356 | 0.722 |
| AQ38 | -0.084 | 0.047 | -1.770 | 0.077 |
| AQ39 | 0.047 | 0.049 | 0.955 | 0.340 |
| AQ40 | 0.367 | 0.054 | 6.835 | 0.000 |
| AQ41 | -0.049 | 0.050 | -0.981 | 0.326 |
| AQ42 | 0.063 | 0.053 | 1.187 | 0.235 |
| AQ43 | 0.072 | 0.052 | 1.371 | 0.170 |
| AQ44 | 0.050 | 0.040 | 1.240 | 0.215 |
| AQ45 | 0.000 | 0.046 | 0.000 | 1.000 |
| AQ46 | 0.097 | 0.049 | 1.992 | 0.046 |
| AQ47 | 0.059 | 0.039 | 1.523 | 0.128 |
| AQ48 | 0.102 | 0.062 | 1.663 | 0.096 |
| AQ49 | -0.014 | 0.046 | -0.303 | 0.762 |
| AQ50 | 0.243 | 0.052 | 4.673 | 0.000 |
| ASRS01 | 0.122 | 0.053 | 2.281 | 0.023 |
| ASRS02 | 0.095 | 0.059 | 1.619 | 0.105 |
| ASRS03 | 0.072 | 0.052 | 1.379 | 0.168 |
| ASRS04 | 0.100 | 0.059 | 1.680 | 0.093 |
| **ASRS05** | **0.531** | **0.039** | **13.578** | **0.000** |
| **ASRS06** | **0.762** | **0.029** | **26.523** | **0.000** |
| ASRS07 | 0.346 | 0.049 | 7.057 | 0.000 |
| **ASRS08** | **0.402** | **0.048** | **8.306** | **0.000** |
| ASRS09 | 0.366 | 0.043 | 8.575 | 0.000 |
| ASRS10 | 0.192 | 0.055 | 3.466 | 0.001 |
| ASRS11 | 0.323 | 0.046 | 7.090 | 0.000 |
| ASRS12 | 0.284 | 0.048 | 5.921 | 0.000 |
| **ASRS13** | **0.818** | **0.027** | **29.915** | **0.000** |
| **ASRS14** | **0.479** | **0.036** | **13.283** | **0.000** |
| ASRS15 | 0.116 | 0.047 | 2.487 | 0.013 |
| ASRS16 | 0.220 | 0.044 | 5.017 | 0.000 |
| ASRS17 | 0.345 | 0.048 | 7.141 | 0.000 |
| ASRS18 | 0.239 | 0.049 | 4.835 | 0.000 |
| PDINY01 | 0.097 | 0.053 | 1.836 | 0.066 |
| PDINY02 | 0.069 | 0.068 | 1.020 | 0.308 |
| PDINY03 | 0.068 | 0.051 | 1.323 | 0.186 |
| PDINY04 | 0.097 | 0.060 | 1.627 | 0.104 |
| PDINY05 | 0.206 | 0.062 | 3.331 | 0.001 |
| PDINY06 | -0.014 | 0.036 | -0.371 | 0.711 |
| PDINY07 | 0.006 | 0.044 | 0.143 | 0.886 |
| PDINY08 | -0.053 | 0.055 | -0.968 | 0.333 |
| PDINY09 | 0.168 | 0.052 | 3.231 | 0.001 |
| PDINY10 | 0.242 | 0.053 | 4.603 | 0.000 |
| PDINY11 | -0.125 | 0.071 | -1.754 | 0.079 |
| PDINY12 | 0.018 | 0.052 | 0.347 | 0.728 |
| PDINY13 | 0.131 | 0.060 | 2.195 | 0.028 |
| PDINY14 | 0.206 | 0.054 | 3.809 | 0.000 |
| PDINY15 | -0.043 | 0.047 | -0.916 | 0.360 |
| PDINY16 | 0.009 | 0.052 | 0.165 | 0.869 |
| PDINY17 | 0.010 | 0.048 | 0.205 | 0.837 |
| PDINY18 | 0.165 | 0.056 | 2.940 | 0.003 |
| PDINY19 | 0.165 | 0.060 | 2.741 | 0.006 |
| PDINY20 | 0.157 | 0.058 | 2.689 | 0.007 |
| PDINY21 | 0.074 | 0.067 | 1.095 | 0.273 |

Standardised loadings of all AQ-, ASRS- and PDI-items, on each of the 7 factors. Items with standardised loadings of at least 0.4 are reported in bold.
