# Supplementary material for: Delusion-proneness displays comorbidity with traits of autistic-spectrum disorders and ADHD
Source: PLoS One. 2017 May 18;12(5):e0177820. doi: 10.1371/journal.pone.0177820 (PMC5436821; doi:10.1371/journal.pone.0177820)
Supplement: S4 Table — Significant correlations reported in bold (DOCX) [file pone.0177820.s004.docx]

**Delusion-proneness displays comorbidity with traits of Autistic-Spectrum Disorders and ADHD**

**S4 Table.** **Correlations between the different factors from the 7-factor model analysis (full questionnaires)**

|  |  | **Two-Tailed** | | | |
| --- | --- | --- | --- | --- | --- |
|  |  | **Estimate** | **S.E.** | **Est./S.E.** | **p-value** |
| Delusion-proneness with | Social interaction impairment | 0.080 | 0.053 | 1.522 | 0.128 |
|  | **Focus on details** | **0.152** | **0.039** | **3.866** | **0.000** |
|  | **Communication/Social impulsivity** | **0.240** | **0.039** | **6.164** | **0.000** |
|  | Theory of mind | -0.021 | 0.063 | -0.337 | 0.736 |
|  | **Inattention** | **0.176** | **0.047** | **3.788** | **0.000** |
|  | **Hyperactivity** | **0.244** | **0.037** | **6.631** | **0.000** |
| Hyperactivity with | Social interaction impairment | 0.074 | 0.053 | 1.394 | 0.163 |
|  | **Focus on details** | **0.099** | **0.042** | **2.335** | **0.020** |
|  | **Communication/Social impulsivity** | **0.303** | **0.040** | **7.506** | **0.000** |
|  | Theory of mind | 0.026 | 0.053 | 0.488 | 0.625 |
|  | **Inattention** | **0.199** | **0.043** | **4.662** | **0.000** |
| Inattention with | **Social interaction impairment** | **0.211** | **0.053** | **3.996** | **0.000** |
|  | **Focus on details** | **-0.083** | **0.041** | **-2.022** | **0.043** |
|  | **Communication/Social impulsivity** | **0.203** | **0.042** | **4.801** | **0.000** |
|  | Theory of mind | 0.064 | 0.050 | 1.300 | 0.194 |
| Theory of mind with | **Social interaction impairment** | **0.358** | **0.041** | **8.646** | **0.000** |
|  | Focus on details | -0.043 | 0.054 | -0.789 | 0.430 |
|  | **Communication/Social impulsivity** | **0.098** | **0.046** | **2.138** | **0.033** |
| Communication/Social impulsivity with | **Social interaction impairment** | **0.106** | **0.048** | **2.235** | **0.025** |
|  | **Focus on details** | **0.115** | **0.046** | **2.537** | **0.011** |
| Focus on details with | Social interaction impairment | 0.052 | 0.054 | 0.971 | 0.331 |

Significant correlations reported in bold
