# Supplementary material for: Delusion-proneness displays comorbidity with traits of autistic-spectrum disorders and ADHD
Source: PLoS One. 2017 May 18;12(5):e0177820. doi: 10.1371/journal.pone.0177820 (PMC5436821; doi:10.1371/journal.pone.0177820)
Supplement: S5 Table — Standardised loadings of all the selected AQ-, ASRS- and PDI-items, on each of the 5 factors. Items with standardised loadings of at least 0.4 are reported in bold. (DOCX) [file pone.0177820.s005.docx]

**Delusion-proneness displays comorbidity with traits of Autistic-Spectrum Disorders and ADHD**

**S5 Table. 5-factor model (truncated questionnaires)**

|  | | **Two-Tailed** | | | | | | | |
| --- | --- | --- | --- | --- | --- | --- | --- | --- | --- |
| **Factor 1** | | **Estimate** | | **S.E.** | | **Est./S.E.** | | **p-value** | |
| AQ01 | | 0.207 | | 0.060 | | 3.461 | | 0.001 | |
| AQ02 | | 0.258 | | 0.054 | | 4.772 | | 0.000 | |
| AQ08 | | 0.376 | | 0.069 | | 5.415 | | 0.000 | |
| AQ09 | | 0.245 | | 0.070 | | 3.477 | | 0.001 | |
| AQ16 | | 0.250 | | 0.056 | | 4.474 | | 0.000 | |
| **AQ20** | | **0.689** | | **0.056** | | **12.317** | | **0.000** | |
| **AQ22** | | **0.700** | | **0.050** | | **13.970** | | **0.000** | |
| AQ25 | | 0.226 | | 0.057 | | 3.969 | | 0.000 | |
| **AQ26** | | **0.683** | | **0.052** | | **13.081** | | **0.000** | |
| **AQ35** | | **0.447** | | **0.067** | | **6.663** | | **0.000** | |
| **AQ36** | | **0.562** | | **0.054** | | **10.414** | | **0.000** | |
| AQ41 | | 0.186 | | 0.060 | | 3.101 | | 0.002 | |
| **AQ42** | | **0.519** | | **0.050** | | **10.365** | | **0.000** | |
| **AQ45** | | **0.784** | | **0.044** | | **17.912** | | **0.000** | |
| ASRS2 | | 0.143 | | 0.045 | | 3.187 | | 0.001 | |
| ASRS3 | | 0.034 | | 0.036 | | 0.924 | | 0.355 | |
| ASRS4 | | 0.045 | | 0.040 | | 1.131 | | 0.258 | |
| ASRS5 | | -0.004 | | 0.030 | | -0.126 | | 0.899 | |
| ASRS6 | | 0.006 | | 0.024 | | 0.243 | | 0.808 | |
| ASRS7 | | 0.007 | | 0.040 | | 0.181 | | 0.856 | |
| ASRS9 | | 0.205 | | 0.041 | | 4.997 | | 0.000 | |
| ASRS10 | | -0.012 | | 0.037 | | -0.308 | | 0.758 | |
| ASRS13 | | 0.090 | | 0.036 | | 2.504 | | 0.012 | |
| ASRS14 | | 0.142 | | 0.042 | | 3.351 | | 0.001 | |
| ASRS16 | | -0.107 | | 0.047 | | -2.256 | | 0.024 | |
| ASRS17 | | -0.010 | | 0.030 | | -0.342 | | 0.732 | |
| ASRS18 | | -0.012 | | 0.032 | | -0.380 | | 0.704 | |
| PDINY02 | | 0.072 | | 0.069 | | 1.040 | | 0.298 | |
| PDINY04 | | 0.080 | | 0.069 | | 1.170 | | 0.242 | |
| PDINY05 | | 0.180 | | 0.071 | | 2.531 | | 0.011 | |
| PDINY08 | | 0.038 | | 0.055 | | 0.693 | | 0.488 | |
| PDINY09 | | -0.130 | | 0.065 | | -2.010 | | 0.044 | |
| PDINY12 | | -0.020 | | 0.042 | | -0.469 | | 0.639 | |
| PDINY18 | | -0.046 | | 0.049 | | -0.931 | | 0.352 | |
| PDINY19 | | 0.098 | | 0.073 | | 1.348 | | 0.178 | |
| PDINY20 | | -0.078 | | 0.056 | | -1.399 | | 0.162 | |
|  | | **Two-Tailed** | | | | | | | |
| **Factor 2** | | **Estimate** | | **S.E.** | | **Est./S.E.** | | **p-value** | |
| AQ01 | | 0.026 | | 0.056 | | 0.472 | | 0.637 | |
| AQ02 | | -0.169 | | 0.054 | | -3.134 | | 0.002 | |
| AQ08 | | 0.137 | | 0.065 | | 2.102 | | 0.036 | |
| AQ09 | | -0.256 | | 0.064 | | -4.021 | | 0.000 | |
| AQ16 | | -0.012 | | 0.046 | | -0.253 | | 0.800 | |
| AQ20 | | -0.130 | | 0.071 | | -1.820 | | 0.069 | |
| AQ22 | | 0.176 | | 0.075 | | 2.358 | | 0.018 | |
| AQ25 | | -0.069 | | 0.054 | | -1.274 | | 0.203 | |
| AQ26 | | 0.183 | | 0.072 | | 2.546 | | 0.011 | |
| AQ35 | | -0.011 | | 0.057 | | -0.198 | | 0.843 | |
| AQ36 | | 0.006 | | 0.040 | | 0.149 | | 0.882 | |
| AQ41 | | -0.192 | | 0.059 | | -3.279 | | 0.001 | |
| AQ42 | | -0.093 | | 0.059 | | -1.596 | | 0.110 | |
| AQ45 | | -0.001 | | 0.040 | | -0.015 | | 0.988 | |
| **ASRS2** | | **0.630** | | **0.034** | | **18.369** | | **0.000** | |
| **ASRS3** | | **0.597** | | **0.036** | | **16.662** | | **0.000** | |
| **ASRS4** | | **0.784** | | **0.034** | | **23.332** | | **0.000** | |
| ASRS5 | | 0.255 | | 0.036 | | 7.017 | | 0.000 | |
| ASRS6 | | -0.013 | | 0.023 | | -0.565 | | 0.572 | |
| ASRS7 | | 0.315 | | 0.038 | | 8.394 | | 0.000 | |
| ASRS9 | | 0.281 | | 0.042 | | 6.708 | | 0.000 | |
| **ASRS10** | | **0.495** | | **0.035** | | **13.946** | | **0.000** | |
| ASRS13 | | 0.029 | | 0.025 | | 1.141 | | 0.254 | |
| ASRS14 | | 0.111 | | 0.038 | | 2.912 | | 0.004 | |
| ASRS16 | | 0.019 | | 0.035 | | 0.547 | | 0.584 | |
| ASRS17 | | 0.082 | | 0.063 | | 1.312 | | 0.189 | |
| ASRS18 | | 0.175 | | 0.055 | | 3.166 | | 0.002 | |
| PDINY02 | | -0.231 | | 0.073 | | -3.159 | | 0.002 | |
| PDINY04 | | 0.034 | | 0.050 | | 0.670 | | 0.503 | |
| PDINY05 | | 0.106 | | 0.058 | | 1.811 | | 0.070 | |
| PDINY08 | | -0.141 | | 0.070 | | -2.023 | | 0.043 | |
| PDINY09 | | 0.047 | | 0.041 | | 1.153 | | 0.249 | |
| PDINY12 | | -0.089 | | 0.055 | | -1.633 | | 0.103 | |
| PDINY18 | | 0.142 | | 0.052 | | 2.720 | | 0.007 | |
| PDINY19 | | -0.006 | | 0.042 | | -0.150 | | 0.880 | |
| PDINY20 | | 0.045 | | 0.054 | | 0.832 | | 0.406 | |

|  | **Two-Tailed** | | | |
| --- | --- | --- | --- | --- |
| **Factor 3** | **Estimate** | **S.E.** | **Est./S.E.** | **p-value** |
| AQ01 | -0.132 | 0.059 | -2.217 | 0.027 |
| AQ02 | -0.015 | 0.055 | -0.264 | 0.792 |
| AQ08 | 0.032 | 0.061 | 0.517 | 0.605 |
| AQ09 | -0.046 | 0.057 | -0.808 | 0.419 |
| AQ16 | 0.084 | 0.057 | 1.476 | 0.140 |
| AQ20 | 0.064 | 0.066 | 0.974 | 0.330 |
| AQ22 | -0.324 | 0.072 | -4.510 | 0.000 |
| AQ25 | 0.039 | 0.059 | 0.654 | 0.513 |
| AQ26 | -0.305 | 0.060 | -5.048 | 0.000 |
| AQ35 | 0.025 | 0.063 | 0.405 | 0.685 |
| AQ36 | 0.076 | 0.061 | 1.243 | 0.214 |
| AQ41 | -0.074 | 0.059 | -1.257 | 0.209 |
| AQ42 | 0.014 | 0.051 | 0.283 | 0.778 |
| AQ45 | 0.036 | 0.052 | 0.697 | 0.486 |
| ASRS2 | 0.023 | 0.033 | 0.694 | 0.488 |
| ASRS3 | -0.025 | 0.034 | -0.747 | 0.455 |
| ASRS4 | -0.089 | 0.041 | -2.184 | 0.029 |
| **ASRS5** | **0.477** | **0.037** | **12.768** | **0.000** |
| **ASRS6** | **0.769** | **0.035** | **21.890** | **0.000** |
| ASRS7 | 0.268 | 0.041 | 6.522 | 0.000 |
| ASRS9 | 0.215 | 0.048 | 4.443 | 0.000 |
| ASRS10 | 0.138 | 0.046 | 3.036 | 0.002 |
| **ASRS13** | **0.830** | **0.030** | **27.502** | **0.000** |
| **ASRS14** | **0.408** | **0.043** | **9.553** | **0.000** |
| ASRS16 | 0.108 | 0.075 | 1.448 | 0.148 |
| ASRS17 | 0.148 | 0.109 | 1.359 | 0.174 |
| ASRS18 | 0.096 | 0.085 | 1.130 | 0.258 |
| PDINY02 | -0.022 | 0.064 | -0.345 | 0.730 |
| PDINY04 | -0.001 | 0.052 | -0.020 | 0.984 |
| PDINY05 | 0.052 | 0.056 | 0.938 | 0.348 |
| PDINY08 | -0.073 | 0.068 | -1.076 | 0.282 |
| PDINY09 | 0.113 | 0.057 | 1.990 | 0.047 |
| PDINY12 | 0.035 | 0.051 | 0.682 | 0.495 |
| PDINY18 | 0.110 | 0.060 | 1.846 | 0.065 |
| PDINY19 | -0.005 | 0.050 | -0.107 | 0.915 |
| PDINY20 | 0.089 | 0.061 | 1.468 | 0.142 |

|  | **Two-Tailed** | | | |
| --- | --- | --- | --- | --- |
| **Factor 4** | **Estimate** | **S.E.** | **Est./S.E.** | **p-value** |
| AQ01 | 0.075 | 0.063 | 1.183 | 0.237 |
| AQ02 | -0.055 | 0.054 | -1.019 | 0.308 |
| AQ08 | -0.182 | 0.086 | -2.100 | 0.036 |
| AQ09 | 0.227 | 0.075 | 3.035 | 0.002 |
| AQ16 | 0.112 | 0.061 | 1.842 | 0.066 |
| AQ20 | -0.090 | 0.075 | -1.196 | 0.232 |
| AQ22 | 0.074 | 0.057 | 1.295 | 0.195 |
| AQ25 | 0.044 | 0.060 | 0.732 | 0.464 |
| AQ26 | 0.115 | 0.067 | 1.716 | 0.086 |
| AQ35 | 0.062 | 0.070 | 0.892 | 0.372 |
| AQ36 | -0.163 | 0.076 | -2.151 | 0.031 |
| AQ41 | 0.162 | 0.064 | 2.540 | 0.011 |
| AQ42 | -0.028 | 0.051 | -0.558 | 0.577 |
| AQ45 | -0.127 | 0.074 | -1.724 | 0.085 |
| ASRS2 | -0.081 | 0.042 | -1.921 | 0.055 |
| ASRS3 | 0.069 | 0.039 | 1.804 | 0.071 |
| ASRS4 | -0.027 | 0.030 | -0.898 | 0.369 |
| ASRS5 | 0.086 | 0.046 | 1.859 | 0.063 |
| ASRS6 | 0.132 | 0.050 | 2.633 | 0.008 |
| ASRS7 | -0.008 | 0.041 | -0.202 | 0.840 |
| ASRS9 | 0.108 | 0.045 | 2.417 | 0.016 |
| ASRS10 | 0.066 | 0.039 | 1.686 | 0.092 |
| ASRS13 | 0.060 | 0.046 | 1.305 | 0.192 |
| ASRS14 | 0.009 | 0.035 | 0.260 | 0.795 |
| ASRS16 | 0.119 | 0.048 | 2.507 | 0.012 |
| ASRS17 | -0.026 | 0.027 | -0.963 | 0.335 |
| ASRS18 | -0.081 | 0.043 | -1.897 | 0.058 |
| **PDINY02** | **0.455** | **0.074** | **6.149** | **0.000** |
| **PDINY04** | **0.612** | **0.054** | **11.368** | **0.000** |
| **PDINY05** | **0.552** | **0.060** | **9.273** | **0.000** |
| **PDINY08** | **0.481** | **0.071** | **6.821** | **0.000** |
| **PDINY09** | **0.669** | **0.046** | **14.434** | **0.000** |
| **PDINY12** | **0.654** | **0.053** | **12.342** | **0.000** |
| **PDINY18** | **0.493** | **0.054** | **9.152** | **0.000** |
| **PDINY19** | **0.626** | **0.055** | **11.467** | **0.000** |
| **PDINY20** | **0.582** | **0.057** | **10.260** | **0.000** |

|  | **Two-Tailed** | | | |
| --- | --- | --- | --- | --- |
| **Factor 5** | **Estimate** | **S.E.** | **Est./S.E.** | **p-value** |
| AQ01 | -0.010 | 0.058 | -0.179 | 0.858 |
| AQ02 | 0.121 | 0.063 | 1.921 | 0.055 |
| AQ08 | -0.183 | 0.080 | -2.296 | 0.022 |
| AQ09 | 0.153 | 0.070 | 2.171 | 0.030 |
| AQ16 | 0.231 | 0.062 | 3.712 | 0.000 |
| AQ20 | 0.078 | 0.082 | 0.953 | 0.341 |
| AQ22 | -0.103 | 0.053 | -1.930 | 0.054 |
| AQ25 | 0.158 | 0.063 | 2.499 | 0.012 |
| AQ26 | 0.017 | 0.025 | 0.678 | 0.498 |
| AQ35 | 0.116 | 0.070 | 1.657 | 0.098 |
| AQ36 | -0.211 | 0.076 | -2.760 | 0.006 |
| AQ41 | 0.179 | 0.062 | 2.883 | 0.004 |
| AQ42 | 0.068 | 0.065 | 1.049 | 0.294 |
| AQ45 | -0.110 | 0.075 | -1.475 | 0.140 |
| ASRS2 | -0.003 | 0.029 | -0.092 | 0.926 |
| ASRS3 | 0.136 | 0.047 | 2.886 | 0.004 |
| ASRS4 | 0.057 | 0.039 | 1.456 | 0.146 |
| ASRS5 | 0.014 | 0.032 | 0.447 | 0.655 |
| ASRS6 | 0.030 | 0.035 | 0.871 | 0.384 |
| ASRS7 | 0.046 | 0.038 | 1.219 | 0.223 |
| ASRS9 | 0.125 | 0.043 | 2.896 | 0.004 |
| ASRS10 | 0.059 | 0.039 | 1.503 | 0.133 |
| ASRS13 | -0.018 | 0.027 | -0.644 | 0.520 |
| ASRS14 | 0.113 | 0.044 | 2.574 | 0.010 |
| **ASRS16** | **0.471** | **0.048** | **9.851** | **0.000** |
| **ASRS17** | **0.756** | **0.058** | **12.928** | **0.000** |
| **ASRS18** | **0.558** | **0.051** | **10.988** | **0.000** |
| PDINY02 | 0.148 | 0.079 | 1.863 | 0.062 |
| PDINY04 | -0.010 | 0.057 | -0.184 | 0.854 |
| PDINY05 | 0.015 | 0.048 | 0.314 | 0.754 |
| PDINY08 | 0.049 | 0.064 | 0.757 | 0.449 |
| PDINY09 | -0.055 | 0.056 | -0.971 | 0.332 |
| PDINY12 | -0.141 | 0.069 | -2.036 | 0.042 |
| PDINY18 | -0.025 | 0.051 | -0.500 | 0.617 |
| PDINY19 | 0.099 | 0.064 | 1.555 | 0.120 |
| PDINY20 | -0.023 | 0.055 | -0.417 | 0.676 |

Standardised loadings of all the selected AQ-, ASRS- and PDI-items, on each of the 5 factors. Items with standardised loadings of at least 0.4 are reported in bold.
