# Supplementary material for: Delusion-proneness displays comorbidity with traits of autistic-spectrum disorders and ADHD
Source: PLoS One. 2017 May 18;12(5):e0177820. doi: 10.1371/journal.pone.0177820 (PMC5436821; doi:10.1371/journal.pone.0177820)
Supplement: S6 Table — (DOCX) [file pone.0177820.s006.docx]

**Delusion-proneness displays comorbidity with traits of Autistic-Spectrum Disorders and ADHD**

**S6 Table. Significant correlations between the different factors from the 5-factor model analysis**

|  |  | **Two-Tailed** | | | |
| --- | --- | --- | --- | --- | --- |
|  |  | **Estimate** | **S.E.** | **Est./S.E.** | **p-value** |
| PDI with | AQ | 0.212 | 0.051 | 4.145 | 0.000 |
|  | ASRS1 | 0.160 | 0.046 | 3.454 | 0.001 |
|  | ASRS2 | 0.258 | 0.048 | 5.357 | 0.000 |
|  | ASRS3 | 0.235 | 0.049 | 4.760 | 0.000 |
| AQ with | ASRS1 | 0.249 | 0.052 | 4.787 | 0.000 |
|  | ASRS2 | 0.166 | 0.063 | 2.612 | 0.009 |
|  | ASRS3 | 0.122 | 0.056 | 2.173 | 0.030 |
| ASRS1 with | ASRS2 | 0.285 | 0.044 | 6.409 | 0.000 |
|  | ASRS3 | 0.170 | 0.056 | 3.022 | 0.003 |
| ASRS3 with | ASRS2 | 0.398 | 0.088 | 4.525 | 0.000 |
