# Supplementary material for: Delusion-proneness displays comorbidity with traits of autistic-spectrum disorders and ADHD
Source: PLoS One. 2017 May 18;12(5):e0177820. doi: 10.1371/journal.pone.0177820 (PMC5436821; doi:10.1371/journal.pone.0177820)
Supplement: S1 Appendix — (DOCX) [file pone.0177820.s007.docx]

**Delusion-proneness displays comorbidity with traits of Autistic-Spectrum Disorders and ADHD**

**S1 Appendix. Correlation analyses**

PDI yes/no scores and ASRS-inattention scores: r=0.335, p<0.001

PDI yes/no scores and ASRS-hyperactivity/impulsivity scores: r=0.382, p<0.001
