# Supplementary material for: Delusion-proneness displays comorbidity with traits of autistic-spectrum disorders and ADHD
Source: PLoS One. 2017 May 18;12(5):e0177820. doi: 10.1371/journal.pone.0177820 (PMC5436821; doi:10.1371/journal.pone.0177820)
Supplement: S2 Appendix — (DOCX) [file pone.0177820.s008.docx]

**Delusion-proneness displays comorbidity with traits of Autistic-Spectrum Disorders and ADHD**

**S2 Appendix. “Super-healthy” group**

We performed a correlation analysis with a group of “super-healthy” subjects. The reason for constructing a “super-healthy” group was to show that individuals with a possible ADHD symptomatology did not drive our correlational findings. This does not mean that the excluded subjects in this group had ADHD or ASD; only that the chance for having the respective disorder was extremely low in the “super-healthy” group. The part A/B scoring method is only a screening method, a clinical diagnosis requires a full assessment.

In addition, though the number of excluded participants might seem large, it is worth looking at it in more details. Firstly, in the present sample, 16 participants (1.7% of our population) were excluded because they had a score of 32 and above, which is in line with the usual figures (about 1.5%[1]). So when it comes to ASD, excluding 16 participants out of 925 is not so surprising. Secondly, regarding ADHD, we used the screening approach based on the part A/part B scoring method. This method is a pretty crude way of screening as people scoring above 4 on this part range from possibly having ADHD to extremely likely of having ADHD. In our sample, 246 participants (26.6% of our population – 10 were also part of the 16 who scored above 32 on AQ) had a score equal or above 4 on part A (ASRS). Though this is a large score, it is in line with Gau et al’s study[2] that reported that out of their 2284 participants (male and female participants), 64 (2.8%) were “highly likely to have ADHD”, an additional 74 (3.2%) were “probably having ADHD”, and another 447 (19.6%) were “possibly having ADHD”. This amounts to 25.6% of their total population ranging from possibly having ADHD to highly likely having ADHD, which is similar to us (since we are interested in tendencies). They did not use the screener approach (“score on part A above 4”) but a more refined scoring approach based on different scorings and threshold. So taking into account the fact that participants we excluded represent not only people who are highly susceptible of having ADHD, but also people who are “possibly” having ADHD, our figure (26.6%) is thus quite similar to theirs (25.6%).

Regarding the factor analysis: we have chosen algorithms that are robust against deviations from normal distribution in the factor analysis, and therefore apply even if the study group does not fully represent a general population.

**References**

1. Christensen DL Braun KV, et al BJ. Prevalence of autism spectrum disorders - autism and developmental disabilities monitoring network, 11 sites, United States, 2012. MMWR Surveill Summ. 2016;65: 1–23. doi:http://dx.doi.org/10.15585/mmwr.ss6503a1

2. Gau SSF, Kessler RC, Tseng W-L, Wu Y-Y, Chiu Y-N, Yeh C-B, et al. Association between sleep problems and symptoms of attention-deficit/hyperactivity disorder in young adults. Sleep. 2007;30: 195–201.
